# Supplementary material for: A quantitative, multimodal wearable bioelectronic device for comprehensive stress assessment and sub-classification
Source: Nat Commun. 2026 Jan 29;17:1150. doi: 10.1038/s41467-025-67747-9 (PMC12858848; doi:10.1038/s41467-025-67747-9)
Supplement: Supplementary file 1 — Supplementary Information [file 41467_2025_67747_MOESM1_ESM.pdf]

Supplementary Information

A quantitative, multimodal wearable bioelectronic device for comprehensive stress assessment and sub-classification

Xiaochang Pei[1,2,#], Anita Ghandehari[1,2,#], Shingirirai Chakoma[1,2,#], Jerome Rajendran[1,2,3,#], Jorge Alfonso Tavares-Negrete[2,4], and Rahim Esfandyarpour[1,2,3,4,*]

[1] Department of Electrical Engineering and Computer Science, University of California, Irvine, CA, 92697, USA

[2] Laboratory for Integrated Nano Bio Electronics Innovation, The Henry Samueli School of Engineering, University of California, Irvine, CA, 92697, USA

[3] Henry Samueli School of Engineering, University of California, Irvine, CA, 92697, USA
[4] Department of Biomedical Engineering, University of California, Irvine, CA, 92697, USA

[#] authors contribute equally.

* Corresponding author: R.E (rahimes@uci.edu)

Supplementary Information Notes:

Supplementary Note. 1 | Mechanical stability of the island-serpentine structure for ECG and GSR electrodes.
Supplementary Note. 2 | ECG and GSR sensor crosstalk study for multi-sensor simultaneous measurement.
Supplementary Note. 3 | Design, Fabrication, and Characterization of MXene/PANI/PEDOT Composite Fabric for PSI Patches in RAP Sensing Applications.

- Design and fabrication of MXene/PANI/PEDOT Composite Fabric.
- Comprehensive Characterization of MXene/PANI/PEDOT Composite.
- Mechanical Stability of the RAP sensor.

Supplementary Note. 4 | Detailed Analysis and Characterization of MSB: Impact of Incubation Periods, Mechanical Characterization, and Integration with Microfluidic Channel.

- Impact of Incubation Periods and Reproducibility of MSB's Electrode Arrays.
- Mechanical characterization of MIP-cortisol sensor.
- Integration and Biocompatibility of MSB’s Electrode Array with Microfluidic Channel for In-Situ Wireless Sweat Analysis.

Supplementary Note. 5 | Analysis of sweat refreshing time and simulations.
Supplementary Note. 6 | Detailed fabrication process and mechanical durability of the PSI patch of the M&M panel.
Supplementary Note. 7 | Detailed design of BLOSDA and WLR for continuous monitoring outside laboratory settings.

- BLOSDA design.
- WLR design.

Supplementary Note. 8 | NFC antenna, readout design and characterization.
Supplementary Note. 9 | Physiological signals qualification and denoising steps.
Supplementary Note. 10 | 1 Dimensional Inception Module (1D-Inception)
Supplementary Note. 11 | Soft Attention (SA) Layer
Supplementary Note. 12 | Complementary explanatory analyses for dataset characterization, interpretability, and validation of Inception-MABFDNN design

Supplementary Information Tables:

Supplementary Table. 1 | Glossary of frequently used abbreviations.
Supplementary Table. 2 | Key stress-related features across different physiological signals as identified in previous studies
Supplementary Table. 3 | Comparison of commercial wearables and our device in stress monitoring features, including PSI/MSB sensing, simultaneous monitoring, stress detection, subtype analysis and their accuracies.
Supplementary Table. 4 | Comparison of existing wearable biosensing systems and our developed device for stress assessment. Features compared include sensed physical and molecular signals, sweat collection methods, sensor reusability, device flexibility, power source, machine learning capability, and stress assessment methodology.

Supplementary Information Figures:

Supplementary Fig. 1 | Biocompatibility Assessment of IP gels with Fibroblast Cells.
Supplementary Fig. 2 | Mechanical stability assessment of the SQC-SAS wearable’s PSI patch: bending and stretching analyses.
Supplementary Fig. 3 | Skin-electrode interface impedance study and SNR analysis of electrodes with various designs.
Supplementary Fig. 4 | SEM images of RAP sensor's e-textile composite.

Supplementary Fig. 5 | Material and Electrical characterization of the conductive e-textile and RAP sensor.
Supplementary Fig. 6 | Mechanical stability of RAP sensor.
Supplementary Fig. 7 | Evaluation of M&M Panel's Mechanical Integrity
Supplementary Fig. 8 | Evaluation of conductivity and mechanical stability of the conductive Ag/SEBS ink.
Supplementary Fig. 9 | Electrochemical validation of MIP preparation on SQC-SAS wearable's MSB electrode arrays.
Supplementary Fig. 10 | Characterization of the SQC-SAS wearable's MSB patch: Incubation time, and sensitivity in artificial sweat.
Supplementary Fig. 11 | Investigating the mechanical stability of SQC-SAS wearable's MSB patch.
Supplementary Fig. 12 | Simulation of the effect of microfluidic inlet number on channel refreshing rate.
Supplementary Fig. 13 | Schematic Illustration of the MSB Fabrication Process.
Supplementary Fig. 14 | Schematic Illustration of the PSI patch Fabrication Process.
Supplementary Fig. 15 | Optical photos of the SQC-SAS wearable Fabrication Process. Step-by-step M&M Fabrication Process and device assembly.
Supplementary Fig. 16 | On-Body Biocompatibility Assessment of SQC-SAS wearable's M&M Panel.
Supplementary Fig. 17 | Detailed circuit schematic of the BLOSDA for the advanced SQC-SAS wearable.
Supplementary Fig. 18 | Detailed circuit schematic of the WLR for the advanced SQC-SAS wearable.
Supplementary Fig. 19 | NFC antenna characterization.
Supplementary Fig. 20 | Detailed representation of cortisol concentration measurements for a subject in response to three different stressors.
Supplementary Fig. 21 | Prolonged wireless and multimodal stress monitoring over 72 hours.
Supplementary Fig. 22 | Overview of physiological signals qualification and denoising workflows.
Supplementary Fig. 23 | Overview of 1D-CNN and 1D-Inception Module structures.
Supplementary Fig. 24 | Chord diagrams that display correlation relationships of features specific to each modality.
Supplementary Fig. 25 | Subject-independent classification performance.
Supplementary Fig. 26 | Subject-dependent classification performance with session-level fine-tuning
Supplementary Fig. 27 | Representative confusion matrices for subject-dependent evaluation.
Supplementary Fig. 28 | Overview of the explanatory analyses steps and results for dataset characterization, interpretability, and validation of Inception-MABFDNN design.

Supplementary Information Videos:

Supplementary Video. 1 | Flexible Microfluidic Channel Infilling Demonstration (Ex-Situ).
Supplementary Video. 2 | Simulation and Ex-Situ Experimental Validation of Flexible Microfluidic Channel Refreshing.
Supplementary Video. 3 | In-Situ (On-Body) Sweat Sample Generation and Channel Infilling.
Supplementary Video. 4 | In-Situ (On-Body) Demonstration of Wireless IP-based Sweat Generation.
Supplementary Video. 5 | In-situ (On-body) On-Demand and Real-Time PSI & MSB Biosignals Collection via SQC-SAS.
Supplementary Video. 6 | Prolonged Continuous In-Situ (On-Body) PSI & MSB Biosignals Collection via WLR Wireless Reader.
Supplementary Video. 7 | In-situ (On-body) On-Demand and Real-Time PSI & MSB Biosignals Collection via SQC-SAS (Without Silicone Case).
Supplementary Video. 8 | Prolonged Continuous In-Situ (On-Body) PSI & MSB Biosignals Collection via WLR Wireless Reader (Without Silicone Case).
Supplementary Video. 9 | Ex-Situ Experimental Demonstration of the Restoration Capability of MSB Patch.
Supplementary Video. 10 | In-Situ (On-Body) Experimental Demonstration of the Restoration Capability of MSB Patch.

Supplementary Video. 11 | SQC-SAS Stress Assessment Capability Demonstration.

Supplementary References

Supplementary Information Notes:

Supplementary Note. 1 | Mechanical stability of the island-serpentine structure for ECG and GSR sensors.

Mechanical stability of sensors is crucial for wearable applications as it ensures the sensor can endure mechanical deformation while maintaining its functionality and performance. We first evaluated the mechanical stability of Design 1 with the island-serpentine structure through a series of cyclic strain tests. Initially, the design was subjected to cyclic strains ranging from 0% to 20% at a frequency of 0.5 Hz using a programmable linear motor while measuring the electrode resistance changes using an impedance spectroscope. The normalized resistance was monitored over 6,000 cycles of stretching, and the results indicated a negligible relative resistance change of less than 2% as illustrated in **Fig. 2E(i)**. This demonstrates the design's ability to maintain electrical performance under repeated mechanical stress. To further illustrate the flexibility of the Design 1 electrode, a cyclic bending test was performed. This test involved bending the electrode within a range of 0% to 30%, simulating the natural curvature of human skin, at a frequency of 0.5 Hz using the linear motor while measuring electrode resistance changes. Remarkably, the relative resistance change remained negligible, also less than 2% after 30,000 bending cycles as shown in **Fig. 2E(ii).** In addition to these mechanical stability tests, the durability of the island-serpentine structure was verified by inspecting the electrode surface for cracks, post-mechanical stability tests. Scanning Electron Microscopy (SEM) images presented in **Supplementary Fig. 2** confirmed the absence of any cracks, thereby substantiating the mechanical resilience of the electrode under continuous cyclic bending and stretching. This comprehensive assessment of mechanical stability highlights the design's suitability for long-term applications requiring sustained mechanical flexibility and durability.

Supplementary Note. 2 | ECG and GSR sensor crosstalk study for multi-sensor simultaneous measurement

Given the ECG and GSR sensors' shared requirement for direct electrode-skin contact for effective data collection, coupled with the conductive nature of the human skin, we implemented careful design strategies to minimize ECG and GSR crosstalk for multi-sensor simultaneous measurements. We performed LTspice simulations to investigate the performance of ECG and GSR sensors when adopting 3-electrode or 2-electrode configurations for ECG. We observed that a 3-electrode ECG sensor's bias applied to the third electrode can induce a current between the ECG and GSR electrodes due to skin conductivity. Our simulations demonstrated a baseline shift in the GSR signal due to ECG-to-GSR sensor current flow through the skin initiated by the ECG's third electrode bias voltage **(Fig. 2I(i))**. Conversely, our findings indicate that this crosstalk can be eliminated in a 2-electrode ECG system by disabling the ECG bias voltage, effectively nullifying current flow to the GSR electrode, even at a minimum skin resistance of 1kΩ **(Fig. 2I(ii))**. Therefore, our PSI panel features a 2-electrode configuration for ECG.

Supplementary Note. 3 | Design, Fabrication, and Characterization of MXene/PANI/PEDOT Composite Fabric for PSI Patches in RAP Sensing Applications

Design and fabrication of MXene/PANI/PEDOT Composite Fabric: The fabrication of the MXene/PANI/PEDOT-based RAP sensor integrated into an electronic textile (e-textile) was a meticulously designed process aimed at developing a highly sensitive and flexible sensor for real-time pulse monitoring. This process involved a systematic sequence of steps, thoughtfully detailed to ensure optimal performance. The foundational material for crafting the e-textile was cotton fabrics, chosen for their lightweight characteristics, cost-effectiveness, flexibility, compressible woven mesh structure, and inherent suitability for wearable applications[1]. To ensure the e-textile's requisite conductivity, MXene, PANI, and PEDOT—proven highly conductive materials—were meticulously applied to a commercially available mesh cotton fabric using the dip-coating method[2]. A compelling aspect of this process is the interaction between MXene nanosheets and cotton textiles, facilitated through hydrogen bonding. This interaction capitalizes on the abundant hydrophilic groups (-O, -OH, and -F) found in MXene, forging hydrogen bonds with the hydroxyl group (-OH) present in cotton textiles[3-5]. The inherent conductivity and substantial surface-to-volume ratio of MXene nanosheets are instrumental in elevating the pressure sensor's sensitivity. They foster the creation of conductive pathways that intricately traverse the fibers within the textile, enabling the seamless transmission of externally applied pressure to the electrical conductance[6]. These woven mesh fabrics were ingeniously transformed into e-textiles designed

specifically for pressure sensing, capitalizing on their three-dimensional foam-like woven mesh structure, which can dramatically change the contact area under various pressures. The narrow gaps between the fibers of the cotton fabric can easily trap the MXene/PANI/PEDOT particles through hydrogen bonding and provide a conductive coating on the fabric as an e-textile. The woven mesh structure of the e-textile fibers allows them to establish extra connections by enlarging the contact area when subjected to external pressure, thereby creating supplementary conductive pathways for electron flow[3,6]. Therefore, the conductance of the e-textile can be used as an indicator of the applied pressure. This innovative concept enabled the sensor to monitor human wrist pulses by measuring the conductance changes in the MXene/PANI/PEDOT coated e-textile.

The interaction between PEDOT and PANI with MXene nanosheets is primarily mediated through π-π bonding and electrostatic forces, facilitated by the positive sulfur groups and negative nitrogen groups[7]. This interplay of molecular forces and charge interactions imparts remarkable properties to the resulting material. To optimize the material properties, we varied the ratios of MXene:PANI:PEDOT during the fabrication of e-textiles. The assembly of the MXene/PANI/PEDOT pressure sensor involved a sandwiching process. These IDEs served the dual purpose of delivering the excitation voltage signal to the e-textile and monitoring the resulting current flow in response to applied pressure[8]. To determine the optimal concentration of MXene/PANI/PEDOT for coating, we empirically investigated the electrical characteristics of the e-textiles (size: 2×1 cm). Various volume ratios (i.e., 1:0:0, 1:0.5:0.5, 1:0.5:1, 1:1:0.5, 0.5:1:1 and 1:1:1) of MXene: PANI: PEDOT were tested. As shown in **Supplementary Fig. 5C**, the slope of the electrical current in the I–V curves (swept from −0.5 to 0.5 V) reveals the conductance of the e-textiles coated with various ratios. The e-textile with the MXene: PANI: PEDOT ratio of 1:1:1 exhibited the highest conductance. We confirmed that the presence of PEDOT and PANI interconnect discrete MXene nanosheets and improve the conductance, offering a better conductive e-textile coating. Based on these results, we fabricated the MXene/PANI/PEDOT e-textile with a 1:1:1 ratio for subsequent experiments.

Comprehensive Characterization of MXene/PANI/PEDOT Composite: Raman spectroscopy played a crucial role in characterizing the synthesized materials. **Supplementary Fig. 5A** illustrates the Raman spectra of PANI, MAX, and MXene powders. The MAX powder displayed distinctive peaks at 148.1, 268.5, and 361.2 cm^{-1}, indicative of the shear and longitudinal vibrations of the Al and Ti atoms, respectively (red curve) [9,10]. Upon removing aluminum from the Ti_3AlC_2 powder, the as-prepared MXene exhibited weak Raman bands in the 200–700 cm^{-1} range (black curve), suggesting structural deterioration and reduced crystallinity [11-13]. Notably, MXene showed unique Raman bands at 262.6, 397.2, and 608.9 cm^{-1}, with a significant signal at 149.3 cm^{-1} due to the formation of Ti_3C_2 [14,15]. Further Raman analysis of the PANI showing characteristic peaks at 1160 and 1185 cm^{-1} (C–H stretching vibration of the benzenoid ring), 1497 cm^{-1} (C=N stretching of the quinoid ring), and 1595 cm^{-1} (C–C stretching of the benzenoid ring) [16,17]. The Raman spectra of both PEDOT and the MXene/PANI/PEDOT composite were analyzed in **Supplementary Fig. 5A**, showing bands consistent with those of pure PEDOT and MXene. This confirms the successful formation of the MXene/PANI/PEDOT hybrid film. Specifically, additional Raman bands were observed at 1360, 1262, 1099, and 985.5 cm^{-1}, corresponding to $C_β$-$C_β$ stretching, $C_α$-$C_α'$ inter-ring stretching, C-O-C deformation, and oxyethylene ring deformation in PEDOT (curves b and c)[18]. The presence of symmetric and asymmetric C=C stretching is indicated by bands at 1425.8 cm^{-1} and 1507.8 cm^{-1} (curves b and c)[19-21]. Notably, the D and G bands of MXene and PANI are masked by the dominant Raman bands of the PEDOT film overlaying the MXene in the MXene/PANI/PEDOT spectrum[13,22].

To gain deeper insights into the material's surface morphology, we employed Scanning Electron Microscopy (SEM). **Supplementary Fig. 4** shows the tightly bonded sheets of the early MAX phase, connected by metallic bonds. After etching and the removal of aluminum from the MAX phase, SEM images of MXene **(Supplementary Fig. 5B)** revealed the successful elimination of aluminum, indicated by the increased interspace among the layers, confirming the effective production of MXene. Furthermore, **Supplementary Fig. 4B** demonstrates the well-distributed PANI particles. Further, MXene/PANI/PEDOT coated fabric SEM showing the proposed composite was coated on the fabric surface **(Supplementary Fig. 5B)**. These findings were consistent with Raman spectroscopy results, corroborating the successful fabrication of the RAP sensor.

Mechanical Stability of the RAP sensor: We further performed the mechanical stability and flexibility characterization of the RAP sensor to demonstrate its suitability for long-term monitoring and wearability. For this we assessed the sensor's output during cyclic pressing and bending tests, as depicted in **(Supplementary Fig. 6A-B)**. In the cyclic pressing test, the sensor was subjected to a constant pressure of 10 kPa for over 50,000 cycles. The relative current change of the sensor displayed almost identical patterns throughout the cyclic tests. Similarly, during the cyclic bending test, the sensor was bent to mimic 30% skin curvature and then released, repeated for over 50,000 cycles. The relative current change during these cycles showed a consistent waveform, as illustrated in **(Fig. 2R)**. The pressure sensor maintained a stable relative current change during the cyclic pressing and bending cycles.

Supplementary Note. 4 | Detailed Analysis and Characterization of MSB: Impact of Incubation Periods, Mechanical Characterization, and Integration with Microfluidic Channel

Impact of Incubation Periods and Reproducibility of MSB's Electrode Arrays: It is essential to emphasize that the duration of incubation periods significantly influences the sensor's response, as shown in **Supplementary Fig. 10A.** Conversely, the volume of the sample demonstrates minimal impact on the sensor's performance **(Fig. 3S).** Reproducibility is a critical aspect of the MSB's electrode arrays for obtaining accurate and reliable results across various electrodes. In our approach, we fabricated five MSB's electrode arrays and evaluated their response to 10 nM cortisol molecules to assess the reproducibility of the fabrication process. As illustrated in **Fig. 3T**, the sensors exhibited highly reproducible responses towards the cortisol biomolecules.

Mechanical characterization of MIP-cortisol sensor: The cortisol sensor was subjected to 30% bending using a linear motor to evaluate its mechanical robustness to withstand cyclic bending **(Fig. 3O)**. During the test, the ECA measurements were recorded in 1 μM Cortisol in PBS solution while taking measurements every 250 bending cycles**.** The sensor exhibited a retention rate of approximately 98-103%, indicating the sensor's ability to maintain its electrochemical performance even after cyclic bending deformations as shown in **Fig. 3P**.

We performed SEM imaging on two sets of electrode samples: one set for before undergoing any bending, and another set after the electrodes had experienced 1000 cycles of bending in order to further assess the mechanical stability of the MIP working electrode and Ag reference electrode to repeated bending. The observed changes in morphology could be attributed to factors like variations in samples, SEM imaging parameters (such as contrast and brightness settings), and the physical impacts of the bending and stretching deformations. Our primary focus was directed towards identifying any signs of surface damage, such as cracking, peeling, or delamination, on the electrode. The SEM images shown in **Fig. 3Q** indicate that the printed composite exhibited no evident cracking, peeling, or delamination from the substrate. However, some minor cracking was discernible on the surface of the working and counter electrodes of the electrochemical sensors. This can be attributed to the composition of the carbon-SEBS ink used, which contains a substantial proportion of small-sized, highly porous materials. This composition renders the composite less capable of stretching when compared to the Ag ink. Further, the MSB electrode array was evaluated for long-term stability over a 21-day period under ambient conditions (**Fig. 3R**). Every seven days, the sensors were removed from storage, and exposed to the same cortisol concentration, and their current response was measured. Following each measurement, the sensors were restored and returned to storage. This cycle was repeated throughout the 21 days, and the results demonstrate a stable sensor response with an RSD of less than 3%. These results highlight the sensor's robustness and suitability for real-world, long-term wearable applications.

Integration and Biocompatibility of MSB's Electrode Array with Microfluidic Channel for In-Situ Wireless Sweat Analysis: The integration of the MSB's electrode array with a microfluidic channel marked a crucial advancement for in-situ wireless sweat analysis. To enhance the efficiency of low-volume sweat sampling (approximately 8.4 μL) and improve the temporal accuracy of wearable sensing, we meticulously engineered a compact and flexible microfluidic channel. This design effectively segregated the sweat

sampling areas from the iontophoresis (IP) gels, ensuring precise separation of these crucial elements within the system. The MSB's electrode array, featuring the integrated microfluidic channel, comprised eight key components, as illustrated in **Supplementary Fig. 13.** Following the electrode printing and MIP polymerization process on the front side of the styrene-ethylene-butylene-styrene (SEBS) substrate, we introduced four hatches (each with a diameter of 0.8 mm) into the SEBS substrate. These hatches played a pivotal role in guiding sweat flow from the microfluidic channel (accumulation layer) at the bottom to the incubation chamber located at the top. On the top side of the SEBS, we established an incubation chamber (with a diameter of 13 mm) by layering prepatterned double-sided medical tape (75 μm thick) and a polyethylene terephthalate (PET) cover layer (13 μm thick) with a single outlet. Similarly, on the bottom side of the SEBS, we fashioned the microfluidic channel for sweat harvesting using layers of prepatterned double-sided medical tape, IP gels, and IP electrodes. The lowest layer of double-sided medical tape served multiple functions, including device attachment to the skin, isolation of the carbachol gel and sweat channels, and sweat accumulation. An illustration of the detailed fabrication process is shown in **Supplementary Fig. 13**. Sweat generated by the NFC powered-IP mechanism is directed into the microfluidic pathway via the specific inlets. It then flows into the sensing reservoir, which holds an approximate sample volume of 8.4 μL. Once the reservoir is filled, the ECA measurement is initiated. Additional sweat secretion, driven by the capillary effect, is then guided towards the outlet**.** Carbachol emerged as the preferred muscarinic agent due to its ability to induce consistent and sustained sweat secretion, attributed in part to its additional nicotinic effects. In contrast, pilocarpine, the conventional sweat-inducing agent used in standard sweat tests and previous wearable systems, only triggered brief sweat production with limited sweat rates from nearby sweat glands[23]. We thoroughly assessed the biocompatibility of Carbachol gel and pilocarpine gel to evaluate their potential for application in our wearable system **(Fig.4Q, 4S, 4T, and Supplementary Fig. 1)**. The biocompatibility assessment involved culturing in direct contact with Carbagel (1% carbachol) and Pilogel (1% pilocarpine), monitoring viability and metabolic activity over seven days. Live/dead assays conducted on days 1, 3, and 7 indicated sustained cell viability of approximately 96% for carbachol. In contrast, pilocarpine exhibited a decrease in cell viability by approximately 80% from day 1 to day 7. Concurrently, metabolic activity was measured over the same period, revealing an increase for carbachol, indicative of cell proliferation in a biocompatible environment. Conversely, pilocarpine showed a decline in metabolic activity throughout the seven-day period. These results demonstrate that carbachol meets biocompatibility criteria, making it suitable for wearable applications.

Supplementary Note. 5 | Analysis of sweat refreshing time and simulations.
Numerical simulations were used to accomplish refreshing time assessments (COMSOL). SOLIDWORKS was used to construct three-dimensional models of various microfluidic designs with the same dimensions as the actual device, which were then imported into COMSOL Multiphysics. The mass transport process was simulated by solving the Stokes equation, which represents an incompressible flow, in combination with the convection-diffusion equation using numerical methods.

$$\nabla\rho = \mu\nabla^2 V \quad (1)$$

$$\frac{\partial C}{\partial t} = \mathrm{D}\nabla^2\, C - V\,\nabla C \quad (2)$$

Where the ρ denotes the pressure, V is the flow velocity, μ is solvent viscosity (water viscosity), C is concentration, and D represents the solute diffusivity[16]. The non-slip boundary condition is applied to all channel walls and a total flow rate of 4 μl min^{-1} is specified for all simulation outputs (graphical and quantitative data), with starting cortisol concentration C_0 fixed at 20 nM and supplied new cortisol concentration C_n preset at 80 nM. Based on our numerical simulations of the optimized geometry design, as depicted in **Supplementary Fig. 12**, we evaluated the performance of different inlet configurations (one to four inlets). The simulation results showed that the configuration with four inlets achieved near 100% replacement of the 20 nM concentration in 300s. In contrast, the models with three, two, and one inlet(s) demonstrated lower refreshment efficiencies, with residual concentrations still present after 300 s. To validate the simulation results regarding the effectiveness of the five-inlet configuration and the refreshment of the old solution, an experimental flow test was also conducted using a microfluidic channel with four inlets, as depicted in **Supplementary Video. 2**. In the experiment, the microfluidic channel was initially filled with a red dye solution, representing a concentration of 20 nM of cortisol. This red dye solution

served as the initial condition for the test. Subsequently, a new blue dye solution was introduced into the channel, assuming a concentration of 80 nM of cortisol. By observing the flow and mixing dynamics in the microfluidic channel, the experiment aimed to evaluate the refreshing time required for the system to effectively remove the red dye concentration and replace it with the blue dye solution. Further, images were captured to examine the microfluidic channel and check for any residual dye from the previous solution. **Fig. 3X** displays the images obtained during the observation. The analysis of these images confirmed that no residual traces of the old solution (red dye) were detected at the end of the experiment. This outcome indicates the successful and complete refreshment of the initial solution.

Supplementary Note. 6 | Detailed fabrication process and mechanical durability of the PSI patch of the M&M panel

As illustrated in **Supplementary Fig. 14,** the fabrication process of the PSI patch's ECG and GSR ultra-thin electrodes, utilizing the CPTP method, begins with laminating a gold (Au)-coated film onto a transfer tape. The Au-coated film is then patterned using a cutter plotter, which precisely cuts the desired electrode patterns based on pre-designed layouts. Following the patterning, the excess film is carefully removed, isolating the patterned electrodes on the transfer tape. The patterned film is subsequently laminated onto Tegaderm film, a flexible substrate which can be stretched up to 450 %, comparable to SEBS **(Supplementary Fig. 7A).** This ensures the electrodes are securely attached and provided with necessary mechanical support. Finally, the device is flipped, and the transfer tape is removed, leaving the patterned electrodes firmly attached to the Tegaderm film. For the RAP sensor, the fiber and bottom insulation layer for the RAP sensor are patterned using similar approaches as the ECG and GSR electrodes. These components are then assembled onto the bottom layer of the patch, forming the structural foundation of the RAP sensor. Next, the patch is flipped, and the top electrode and insulation layer are patterned, ensuring they are correctly shaped and positioned. Finally, the top electrode and insulation layer are assembled onto the top of the panel, integrating all layers and electrodes into a cohesive PSI patch of the M&M panel. The optical photos of the fabrication and assembly of the panel are illustrated in **Supplementary Fig. 15.** The PSI patch exhibits remarkable flexibility and can conform to curved surfaces and withstand stretching deformations without damage or delamination **(Supplementary Fig. 7B-C)**. Additional testing involving twisting, bending, and stretching on human skin further confirmed the panel's durability **(Fig. 2H).** This mechanical durability is crucial for the reliable performance of the physiological stress indicators integrated within the patch.

Supplementary Note. 7 | Detailed design of BLOSDA and WLR for continuous monitoring outside laboratory settings

BLOSDA design: To maximize user comfort and circumvent challenges associated with battery use, such as recharging necessities, biohazard risks, thickness, and rigidity, the BLOSDA operates battery-free. This was accomplished through the integration of an NFC transponder and a flexible receiver antenna into the BLOSDA. The NFC front-end equivalent circuit consists of the NFC transponder (tag), matching capacitor, and antenna **(Supplementary Fig. 19A).** The NFC transponder, adhering to the ISO/IEC 14443 standard for inductive coupling, wirelessly harvests energy from the RF field generated by the NFC reader antenna, simultaneously transmitting data. The harvested energy powers all electronic components within the BLOSDA system and M&M panel. In contrast to conventional wireless protocols such as Bluetooth[23] and Wi-Fi[24], NFC distinguishes itself by offering the dual capabilities of wireless data transfer and power transmission. This unique feature obviates the necessity for battery-dependent devices and further distinguishes NFC by its diminished power consumption in comparison to established wireless counterparts because of its short-range nature and lower operating frequency[25]. Consequently, NFC facilitates an automated mode of communication characterized by superior energy efficiency, reducing user intervention for functional operation. This attribute substantially enhances the safety and convenience attributes of our wearable health monitoring system. Moreover, the MSB and PSI signals data are transmitted from the BLOSDA to the WLR for signal reconstruction and stress feature extraction. The system incorporates an integrated microcontroller unit (MCU) and distinct analog front-end (AFE) circuits, designed to transduce raw signals from the M&M panel into digitized form for transmission to the reader. While the AFE circuits are sensor-specific, they share a common MCU and NFC transducer. Integrated analog-to-digital converters (ADCs) within the ICs transform voltage signals from varied AFE circuits into

digital data. To achieve bidirectional, real-time data transmission from the M&M panel's all sensors, we proposed a memory buffer with quick read-write speeds. Here, the NFC transponder's Static Random-Access Memory (SRAM) functions as a data buffer due to its superior read-write speed compared to the electrically erasable programmable read-only memory (EEPROM) (MCU write operation takes < 4.5 ms for 16-byte data in EEPROM, but < 0.4 ms in SRAM). The SRAM was segmented into five partitions, with each assigned to one sensor's data. The BLOSDA's MCU compiles data from different sensor-associated AFE circuits during the measurement process, storing sensor data in corresponding SRAM addresses. The WLR's MCU then retrieves the SRAM-stored data via inductive coupling with the BLOSDA's NFC antenna, analyzes it, and writes command directives to the SRAM to configure the BLOSDA AFE ICs and regulate power consumption. Subsequently, the BLOSDA's MCU initiates a new cycle, responding to SRAM-stored commands (On or Idle/sleep functions), and continues data collection at ML-determined rates and durations after every constant time period (tp, in the millisecond range). The data throughput will be computed using the formula: throughput = SRAM size / tp (in the millisecond range). Additionally, the BLOSDA system includes an integrated IP current source, enabling innovative wireless NFC-powered iontophoresis for on-demand sweat stimulation. This proposed workflow promises continuous, real-time, simultaneous, user-free, autonomous, power-efficient, and intelligent data collection from multiple sensors for five distinct MSBs and PSIs. Detailed circuit schematics of the BLOSDA are illustrated in **Supplementary Fig. 17**. Besides, the BLOSDA can be interfaced with external electrodes via an FPC cable, allowing subjects greater freedom of movement during extended measurement periods without the need for pressing the back-to-back ECG electrode.

WLR design: The primary functions of the WLR include wireless power transmission to the BLOSDA and reading data from the BLOSDA, facilitated by utilizing an NFC reader and a flexible transmitter antenna. The collected data is stored in a microSD card for further processing. Thus, the WLR was used for continuous monitoring outside laboratory settings by reading data during daily activities while storing it in a microSD card for further analysis. An MCU enables the time sequential collection of data through the NFC reader and storage to a microSD card. The MCU employs two synchronous serial ports (SSPs) configured as serial peripheral interfaces (SPIs) to communicate with both the NFC reader and the microSD card reader. SPI facilitates high-speed data transmission, ensuring that all data from the BLOSDA is received accurately and in the correct chronological sequence. To conserve battery power, the MCU automatically disables the corresponding SSP when the associated SPI is in an idle state. The detailed circuit schematics of the WLR are illustrated in **Supplementary Fig. 18**. To ensure that all WLR circuitry, antenna, and battery are securely affixed to prevent any potential movement or damage during regular use, we utilized rigid PCBs for circuit fabrication and a custom-designed 3D printed case for packaging. The layer-by-layer assembly of the WLR is illustrated in **Fig. 5F.**

Supplementary Note. 8 | *NFC antenna, readout design, and characterization*

Mechanical deformations such as stretching, twisting, and bending are inherent in wearable applications, and this impacts the antenna performance characteristics like quality (Q) factor, resonant frequency, and impedance[26]. Wearable antennas must withstand these mechanical deformations while maintaining optimum performance e.g. return loss < -10 dB. Flexible substrates such as Polyimide (PI)[27], Polyethylene Terephthalate (PET)[28], PDMS[29], Liquid Crystal Polymer (LCP)[30], and various fabrics and textiles[31] have been deployed in wearable antennas, allowing for adaptation to body contours which enhances wearability and withstand the aforementioned mechanical stresses without compromising the performance of the antenna. Our wearable system uses PI as the antenna substrate due to its lower dielectric constant of 3-4 and dielectric losses (δ) of 0.02[32], which reduces the electromagnetic energy absorbed by the substrate resulting in minimum signal distortion from dielectric losses, thus improving antenna efficiency. While Copper (Cu) and silver (Ag) both exhibit similar conductivity levels (~ 60 MS/m and ~63 MS/m, respectively)[33,34], Cu was preferred as the antenna conductive material due to its lower cost and its compatibility with our preferred FPCB manufacturing process. For our SQC-SAS wearable, the WLR and BLOSDA are positioned on the same hand, thus the receiver and transmitter antennas maintain separation distances less than 0.5 cm. This implies that the wireless power transfer (WPT) efficiency mainly depends on the inductive coupling between the antennas. To maximize the coupling, both antennas were designed

with the same geometry. An initial estimation of the number of antenna coil turns that would result in the target inductance (L) of 1-2 µH was determined using the Grover method[29]:

$$L = \frac{K_1 \mu_0 N^2 d}{1+K_2 p} \tag{3}$$

$$p = \frac{d_{out-d_{in}}}{d_{out+d_{in}}} \tag{4}$$

$$d = \frac{d_{out+d_{in}}}{2} \tag{5}$$

where $\mu_0$is the vacuum permeability, *p* is the fill ratio and *d* is the average diameter of coil. For a square coil K_1 and K_2 = 2.34 and 2.75, respectively.

After this initial estimation, the other parameters like geometry and substrate material were optimized in the Ansys Electronics simulation software. The optimized antenna (28 mm x 16 mm) coil has 6 turns, copper thickness of 35 µm, trace width of 254 µm, and trace gap of 254 µm on 50 µm PI substrate. To mitigate the potential antenna detuning effects resulting from alterations in the antenna's operating environment, the characterization of the tag antenna was conducted on-body by attaching it to the wrist using double sided medical tape, while the reader antenna inside the 3D printed case housing assembled PCBs, battery, and the ferrite sheet. The ferrite sheet was used to suppress the induction of unwanted eddy currents in battery and metallic PCB components which would result in energy loss through heating and generate secondary magnetic fields which alter the primary magnetic field of the antenna. A Nano Vector Network Analyzer (NanoVNA) was used to measure the antenna complex impedance at 13.56 MHz. For the WLR antenna, the measured inductance (1.5 µH), resistance (3.2 Ω), and Q factor (39.8), are shown in **(Fig. 5D(i)).** The measured BLOSDA antenna inductance (1.24 µH), resistance (3.1 Ω), and Q factor (34) **(Fig. 5D(ii)).** Notably, despite the antennas having the same geometry, the difference in the inductances is mainly due to the ferrite sheet's enhanced magnetic permeability. The self-resonance frequencies f_{res} of each antenna were determined from the Smith chart at the frequency where the antenna load impedance switches from inductive to capacitive. To complete the antenna equivalent circuits shown in **Supplementary Fig. 19**, the antenna parasitic capacitances (C_a) were determined by:

$$C_a = \frac{1}{(2\pi f_{res})^2 L_a} \tag{6}$$

The resonant frequency of the tag NFC front-end equivalent circuit is given by:

$$f_0 = \frac{1}{2\pi\sqrt{L_a(C_a + C_{int} + C_{ext})}} \tag{7}$$

where NFC tag internal capacitance C_{int} is NFC tag internal capacitance and C_{ext} is the external tuning capacitor.

The WLR's NFC front-end, consisting of an NFC reader chip, matching network, and antenna **(Supplementary Fig. 19)**, serves a dual purpose: wireless data transmission and power transfer via a matching network from the reader to the antenna which in turn generates an alternating magnetic field that oscillates at 13.56 MHz. To ensure the integrity of data transmission and accurate signal reconstruction, the NFC reader was specifically designed to support all NFC bit rates (212 kbit/s, 424 kbit/s, and 848 kbit/s), adhering to the NFC ISO14443 standard[35]. These bit rates correspond to Q factors of 32, 16, and 8, respectively. The commands of the NFC reader are transmitted within the 12.712 MHz and 14.408 MHz sidebands of the 13.56 MHz carrier frequency, which are determined by the 1.696 MHz BW. The NFC reader was designed to fulfill this BW requirement by employing the lowest possible Q factor (8) to ensure the efficient transmission of all bit rates. The NFC reader antenna matching network comprises of an electromagnetic compatibility (EMC) filter with a cutoff frequency of 21 MHz (which must be greater than the 14.408 MHz upper sideband), an impedance adjusting circuit, and damping resistors which serve to dissipate excess energy and adjust the antenna's Q factor (39.8) to achieve the desired value of 8. The NXP antenna design tool was used to determine the reader matching network based on the optimized antenna geometry. The WLR NFC front-end equivalent circuit was first simulated in ANSYS HFSS, and the performance metrics were the return loss and resonant frequency. The actual values of the equivalent circuit were then populated on the PCB and the RF field measurements were performed using a NanoVNA.

The assessment of WLR NFC front-end performance was ascertained through the antenna return loss (S11), achieving minimum S11 of -28.56 dB at 13.56 MHz indicating that the NFC reader delivers maximum

power to the antenna at the operating frequency **(Fig. 5G).** To achieve efficient antenna performance, it is imperative that the S11 remains below the -10 dB threshold, indicative of a scenario where at least 90% of the antenna input power is transmitted, while the reflected power does not exceed 10%[36]. Furthermore, the NFC tag was tuned to achieve resonance precisely at 13.56 MHz, with the parallel impedance manifesting as purely resistive and peaking at the resonant frequency, as shown in **(Fig. 5G)**. Of notable significance is the evaluation of antenna performance during mechanical stability tests which included bending up to 30% to mimic wrist curvature, as shown in **(Fig. 5H).** Remarkably, incremental bending of the antenna up to 10% did not induce a shift in the resonant frequency. Nevertheless, it did result in a marginal reduction in the maximum power delivered from -28.56 dB to -21.66 dB at 0% (planar) and 10% bending configurations, respectively. It is noteworthy that even under the more pronounced 30% bending condition, which prompted a slight shift in the resonant frequency to 13.86 MHz, the S11 at 13.56 MHz (-12 dB) remained below the -10 dB threshold. These outcomes underscore the robust mechanical stability exhibited by the antenna throughout the assessment, owing to the flexible PI substrate. In light of these evaluations, the strategic placement of both the reader and tag antennas was determined to be on the flat region of the wrist. This choice was made with the specific intent of maintaining a planar orientation, thereby ensuring the maximization of power delivered by the reader antenna precisely at the designated frequency of 13.56 MHz.

Supplementary Note. 9 | Physiological signals qualification and denoising steps

Qualifying the collected signals is a critical step in the analysis of physiological signals obtained from multisensory systems, ensuring that only high-quality signals are utilized in the feature extraction and stress prediction processes. This guarantees that the results of the analysis are reliable and free from noise and artifacts. While qualifying the signals is a crucial step in identifying high-quality signals for analysis, it is equally important to perform appropriate signal preprocessing and denoising techniques, such as noise reduction and baseline correction, on each signal prior to elimination from the analysis process.

ECG qualification and denoising: ECG Qualification and denoising are necessary since this signal is prone to contamination from various sources of noise, including electromyography (EMG) noise, baseline drift, and motion artifacts[37]. To mitigate the effects of these noise sources, the pipeline outlined in **Supplementary Fig. 21A** is employed. Within the ECG denoising and qualification pipeline, first, the ECG signal undergoes standardization to eliminate variations in signal magnitude caused by different recording conditions. The standardization process is performed using the equation:

$$\text{Scaled Signal}_{i} = \frac{\text{Signal}_{i} - \text{Signal}_{\text{mean}}}{\text{Signal}_{\text{std}}} \tag{8}$$

Where Signal_{i} represents each sample point in the recorded ECG signal, $\text{Signal}_{\text{mean}}$, and $\text{Signal}_{\text{std}}$ represent the mean and standard deviation of the recorded ECG signal, respectively. After standardization, implementing a High-Pass Filter (HPF) with an order of 2 and a cutoff frequency of 0.5 Hz, is crucial to eliminate baseline drift[38]. Next, the Stationary Wavelet Transform (SWT) is used to decompose the ECG into its detail and approximation coefficients, with the high-pass filters providing the detail coefficients $(D_1, D_2, \ldots, D_N)$ and the low-pass filters providing the approximation coefficients $(A_1, A_2, \ldots, A_N)$. The Haar wavelet, known for its simplicity, is used as the mother wavelet for transformation, with the decomposition level (N) set at seven for optimal frequency separation[39]. To ensure that the signal length is sufficient for the level of decomposition, symmetric padding is applied to reach the nearest number of samples that is a multiple of 2^{N}. For R-Peaks detection and QRS removal, D_4 is used, as it contains the high-frequency components of the QRS complex and is less affected by motion artifacts[39]. The Pan-Tompkins method[40], which is a widely used and well-established algorithm for detecting R-peaks in ECG signals, is utilized to detect the R-peaks in the signal. The 50 ms before and after each R-peak (totaling 100 ms) is considered as the QRS interval and is removed by assigning 0 to the corresponding samples of detail coefficients D_1 to D_6[39]. Multi-resolution thresholding is applied after the removal of the QRS complexes to determine the maximum and minimum values of the ECG signal for each second of the detail coefficients D_1 to D_7 and the approximate coefficient A_7[41]. The calculated thresholds for each window are then used to identify the range of values within the ECG signal, and all values that fall within

this range are assigned a value of 0. The use of multi-resolution thresholding helps to eliminate any irrelevant or noisy information from the ECG signal, resulting in removing motion artifacts from the ECG signal [41]. Next, the Inverse Stationary Wavelet Transform (ISWT) is applied to the transformed coefficients, and the resulting motion artifact signal is subtracted from the ECG signal [39]. In the final step of ECG signal denoising, the objective is to effectively eliminate high-frequency motion artifacts that have an amplitude comparable to that of P and T waves. This is achieved by first removing the QRS complexes from the motion-artifact-removed ECG signal. Subsequently, the SWT is performed at 7 levels using the Haar wavelet, and all values in the detail coefficients D_1 to D_5, which contain the high-frequency component of the decomposed signal, are assigned to 0. Finally, the ISWT is applied to obtain the denoised ECG signal[39].
After the successful completion of the preprocessing stage, an assessment is conducted on a per-minute basis to determine ECG qualification status. If any minute of the ECG signal exhibits a heart rate below 30 bpm or exceeding 220 bpm, it is considered non-compliant and deemed unqualified (**Supplementary Fig. 21A**).

RAP qualification and denoising: Various noise sources, such as baseline wandering, EMG noise, and motion artifacts, can corrupt the RAP signal[42]. To denoise and qualify the RAP signal, the signal is initially subjected to a standardization process (as described in Equation 1). In the next step, baseline drift is removed using a centered moving average (CMA), as illustrated in Fig. 2b. The optimal sliding window size for this procedure is determined to be half of the RAP sampling rate[43]. At the end of this step, the RAP signal becomes free from baseline drift, denoted as, detrended $_{RAP}$ = raw $_{RAP}$ $- \mathrm{CMA}(\mathrm{raw}_{\mathrm{RAP}})$. To remove motion artifacts and noise, the Hilbert transform ($\mathrm{H}(s)$) is applied to the detrended RAP signal (s), and together, they build the analytic signal ($s + \mathrm{H}(s)$). The instantaneous amplitude ($|s + \mathrm{H}(s)|$), also known as the envelope, is calculated from the analytic signal. The envelope is subsequently smoothed using the CMA method. Finally, the detrended RAP signal is divided by the smoothed envelope to produce a detrended, demodulated, and denoised RAP signal[43].
After processing the RAP signal, an assessment is conducted on a per-minute basis to determine its qualification status. The Aboy++ algorithm [44] is utilized to detect systolic peaks in each minute of the RAP signal. The intervals between these peaks are then used to calculate the average Instantaneous Pulse Rate (IPR) for each minute. If, at any minute, the signal exhibits an IPR below 30 beats per minute (bpm) or exceeds 220 bpm, it is considered non-compliant and thus deemed unqualified (**Supplementary Fig. 21B**).

GSR qualification and denoising: The analysis and interpretation of the phasic and tonic components of GSR can be confounded by various sources of artifacts, including motion artifacts, Ebbecke waves, and high-frequency noise artifacts [45]. To mitigate these issues, a band-pass filter with a frequency range of (0.2-1) Hz is employed to extract the phasic component of GSR from the raw data. All other signal components with a frequency higher than 1 Hz are considered phasic noise and motion artifacts[45]. In addition to the use of frequency-based filters to eliminate motion artifacts and high-frequency noise from the phasic component of the GSR signal, it is important to verify the validity of the phasic values. This is necessary as various factors, such as sensor failure, improper electrode-skin contact, or user interference, can impact the quality of the readings[46]. To ensure the quality of the phasic component, two key steps are taken following the denoising process. Firstly, any portion of the phasic component exceeding 8μS is removed[46,47]. Secondly, the temperature sensor value is used to confirm that the electrodes are worn for an adequate amount of time. Any points where the temperature falls outside the range of (30-40) °C are removed from the signal, along with all data points within 5 seconds of the outlying point[46]. This qualification process helps ensure that only high-quality GSR signals are used for further analysis and interpretation. An overview of the GSR processing pipeline is demonstrated in **Supplementary Fig. 21C**.

ST qualification and denoising: The quality of the ST is assessed by comparing the value of a given point to a valid value range of 30 to 40 ℃, as established from previous research[46]. It should be noted that the temperature measurements in this study are derived from skin temperature, which tends to be lower than core body temperature. As a result, we select a marginally lower range of temperatures as the standard values[46]. If the value of a given point falls outside of this range, it is considered unqualified, indicating that

the recorded ST in that sample is unreliable or contaminated by noise. An overview of the ST qualification step is demonstrated in **Supplementary Fig. 21D**.

Supplementary Note. 10 | 1-Dimensional Inception Module (1D-Inception)
Convolutional Neural Networks (CNNs) are widely used in automatic feature extraction from physiological signals due to their ability to learn temporal representations without requiring handcrafted features. Unlike recurrent architectures, which rely on sequential processing and often struggle with limited datasets, CNNs leverage weight sharing and hierarchical feature extraction, enabling faster convergence and improved learning efficiency in data-scarce scenarios. However, given the multi-scale and multi-resolution characteristics of physiological signals [48] traditional CNN architectures (**Supplementary Fig. 22B**), which operate with a fixed kernel scale at each layer may overlook significant features across multiple scales of signals. Larger kernels are effective for more global information, whereas smaller kernels excel in detecting localized details[49]. Therefore, a more adaptive approach is needed to simultaneously analyze data at different resolutions. To overcome the limitations of traditional CNN layers that use a single filter size, which can overlook significant features across multiple scales of signals, Inception-MABFDNN first feeds each signal in the time and frequency domains to a 1-dimensional Inception module[50] (**Supplementary Fig. 22A**). The Inception module is a key component that employs a combination of filters of different sizes, allowing the model to look at both local and global patterns within each input signal. The Inception module processes inputs in parallel through multiple 1D convolutional kernels (1×1, 1×3, and 1×5) alongside a parallel max pooling pathway within each module, enabling multi-scale feature extraction across different temporal resolutions. Utilizing 1×1 convolutions decreases the network's dimensionality. It effectively lowers the computational complexity that leads to the learning of more robust features, contributing to a decrease in the likelihood of overfitting in deep networks [51].

For a given input signal $X = [x_1, x_2, \dots, x_n]$, the 1D-Inception module processes it through the following four parallel pathways,

$$Y_1 = \mathrm{Conv}_{1\times1}\ (X; W_{1\times1}) \tag{9}$$

$$Y_2 = \mathrm{Conv}_{1\times3}\ (\mathrm{Conv}_{1\times1}\ (X; W_{1\times1\to1\times3}); W_{1\times3}) \tag{10}$$

$$Y_3 = \mathrm{Conv}_{1\times5}\ (\mathrm{Conv}_{1\times1}\ (X; W_{1\times1\to1\times5}); W_{1\times5}) \tag{11}$$

$$Y_4 = \mathrm{Conv}_{1\times1}\ (\mathrm{MaxPool}\ (X)) \tag{12}$$

Where Y_1 is the output of the 1×1 convolution with weights $W_{1\times1}$, and $W_{1\times1\to1\times3}$ are the weights for a 1×1 convolution that acts as a bottleneck layer to reduce dimensions before applying $W_{1\times3}$, which are the weights for the subsequent 1×3 convolution to capture wider temporal features. $W_{1\times1\to1\times5}$ are the weights for a 1×1 convolution that prepares the data with a dimensionality reduction before applying $W_{1\times5}$ and Y_4 is the output of the 1×1 convolution applied to the output of a max pooling operation with a kernel size of 1×3. Each layer with 1×1 convolutions utilizes 4 kernels, while the layers with 1x3 and 1x5 convolutions each employ 8 kernels.

Moreover, each 1D-CNN layer uses a Leaky Rectified Linear Unit (Leaky ReLU) activation function following the convolution operation. This function introduces non-linearity into the model, which is crucial for the network to capture complex patterns in the signals. The Leaky ReLU activation function is defined for each element c_i in the output vector C resulting from the 1D-convolution operation as follows:

$$Leaky\ ReLU = max(0.01c_i, c_i) \tag{13}$$

Finally, the outputs from each path of the 1D-inception module are concatenated along the channel dimension to form a unified feature vector, Y, capturing a comprehensive representation of the input signal[51]:

$$Y = \text{Concat}\,(Y_1, Y_2, Y_3, Y_4) \tag{14}$$

Within Inception-MABFDNN, each physiological signal undergoes feature extraction through a dedicated branch. In each branch, two sub-branches are developed: one focuses on extracting temporal features from the original input signal window, and the other is dedicated to extracting spectral features from the Fast Fourier Transform (FFT) of the input signal window. Both sub-branches are founded upon the sequence of three stacked 1D-Inception modules. Within this stack, each module's CNN layers are consistently configured with a stride of 1 and 'same' padding. The output from each sub-branch is fed into an average pooling layer, which has a kernel size and stride equal to the sampling rate of the signal that the sub-branch is dedicated to. This approach ensures a consistent vector size across all signals sub-branches, which is necessary for subsequent analysis. At the end of this segment, the outputs from all branches are concatenated along the channel dimension. The outputs of the concatenated layer are fed into the Soft Attention (SA) layer, which is described in detail in the **Supplementary Note. 11**.

Supplementary Note. 11 | Soft Attention (SA) Layer

Since different sets of extracted features from ECG, GSR, PPG, and ST signals have different degrees of impact on stress detection, the soft attention mechanism, introduced in [52] can be leveraged to weigh the significance of different features, thereby directing the model to focus on the most pertinent set of features for stress detection. Here, we explained the implementation details of applying attention mechanisms to the extracted features. The concatenated output of 1D-Inception-based branches (**Supplementary Note. 10**) can be represented as follows:

$$K = [K_1, K_2, \dots, K_n] \tag{15}$$

This feature vector is then fed to the following attention layer:

$$u_t = \tanh(\omega \cdot K_t + b) \qquad t \in [1, n] \tag{16}$$

$$\alpha_t = \frac{\exp(u_t^T u)}{\sum_{t=1}^{T} \exp\,(u_t^T u)} \tag{17}$$

$$S = \sum_{t=1}^{n} \alpha_t K_t \tag{18}$$

In this attention mechanism, each K_t is inputted to a fully connected neural network with ω as the weight matrix and b as the bias vector. This fully connected neural network is equipped with a hyperbolic tangent (tanh) activation function to generate u_t as its output. The transposition of the output u_t is multiplied by u, a trainable parameter vector, to get the alignment coefficient of attention [52]. Then, a SoftMax function is used to normalize α_t. After that, we compute the vector representation S as a weighted sum of all features. The feature representation S along with the cortisol concentration is passed through a three-layer fully connected neural network (FCNN), which is responsible for generating the final prediction, with layer sizes of 64, 8, and a final output layer containing either 1 neuron for binary stress vs. rest classification or 6 neurons for stress subclassification. The initial two layers utilize the Leaky ReLU activation function for non-linear transformation, whereas the final layer adopts a Sigmoid activation function for binary classification and SoftMax activation function for stress subclassification. During the training process of the Inception-MABFDNN, the Adam optimizer [53] and the cross-entropy loss function are utilized.

Supplementary Note. 12 | Complementary explanatory analyses for dataset characterization, interpretability, and validation of Inception-MABFDNN design

The first step in our analysis was signal preprocessing, including noise reduction and baseline correction **(Supplementary Fig. 28A)**. This step ensured that only high-quality signals were retained for feature extraction and stress prediction, thereby minimizing artifacts and enhancing accuracy **(Supplementary Note. 9; Supplementary Fig. 20).** For dataset characterization and to validate our Inception-MABFDNN design, we focused on specific features in each signal that vary under stress. This approach offers a more easily visualized and interpretable means of examining how each signal's stress-related variations relate to one another. Therefore, we extracted a set of established stress-related features to gain explanatory insight into the collected physiological signals and the cortisol biomarker. The principal characteristics of these signals, which form the basis of stress-related feature extraction, are illustrated in **Supplementary Fig. 28A.**

Stress-Related Features in Physiological Signals. Each heartbeat on an ECG trace is characterized by three primary components: the P wave, the QRS complex, and the T wave. These elements correspond to the electrical impulses that drive the heart's rhythmic contractions. Additionally, the R–R interval, denoting the duration between consecutive ventricular contractions, is an essential feature of ECG recordings from which heart rate (HR) is derived. Variation in the R–R intervals provides insight into the heart's rhythm and rate, offering an indirect measure of the stress response[65]. Galvanic skin response (GSR) reflects fluctuations in the skin's electrical properties due to sweat secretion associated with emotional arousal and stress. The GSR signal has two distinct components: a tonic component known as the skin conductance level (SCL) and a phasic component known as the skin conductance response (SCR)[66]. SCL is a slowly varying baseline level of skin conductance over extended periods. In contrast, SCR represents rapid, transient conductance fluctuations tied to specific events or stimuli, and these phasic responses are highly sensitive to stress-induced changes[67]. Key characteristics of the SCR phasic component (illustrated in **Supplementary Fig. 28A**) include: the onset, marking the beginning of a response; the peak amplitude (Peak Amp.), indicating the maximum conductance level reached; the rise time (SCR RiseT), the duration from SCR onset to peak; and the recovery time (SCR RecT), the period for the SCR to return to baseline after the peak. Another important feature is the offset, which represents the end of the SCR's response to a stimulus[67]. Together, these parameters define event-related SCR peaks and are essential for analyzing the autonomic nervous system's response to stress. We also examined the radial arterial pulse (RAP) waveform, a non-invasive measure that captures cardiovascular responses to stressors by detecting subtle pressure changes over the cardiac cycle. The RAP waveform is characterized by features such as the pulse onset and offset, the systolic peak (sp), the diastolic peak (dp), and the dicrotic notch (dn)[68]. Further details on all hand-crafted stress indicator features are provided in **Supplementary Table. 2.** For feature extraction, each signal was segmented into 5-second windows, and the corresponding stress-related features were computed from each window.

Multimodal Feature Interrelatedness Analysis **(Correlation Analysis).** To evaluate the interrelatedness of the signals and ensure that each sensor modality adds unique information, we calculated Pearson correlation coefficients (PCC) among all extracted stress-related features. For each subject, correlations between features from different physiological signals were consistently low (**Supplementary Fig. 28B**), and correlations between any physiological feature and cortisol concentration were below 0.1. These uniformly low correlation values indicate that each modality captures largely complementary (non-redundant) aspects of the stress response. This finding supports the need for a truly multimodal approach, leveraging ECG, RAP, GSR, ST, and cortisol together, for robust stress detection. Our Inception-MABFDNN architecture was designed with this principle in mind, to integrate information from all modalities.

Feature Space Visualization with t-SNE. We further examined whether the chosen signals and features encode discriminative patterns between rest and stress conditions by visualizing the feature space using t-distributed stochastic neighbor embedding (t-SNE). Projecting the high-dimensional stress-related feature vectors into two dimensions with t-SNE revealed well-separated clusters corresponding to stress versus non-stress states (**Supplementary Fig. 28C**). This visualization provides intuitive confirmation of the discriminatory power of our multimodal signals, supporting their validity as effective markers for stress detection.

Traditional Machine Learning Model- Training and Feature Importance Analysis. To quantify and visualize the heterogeneous (non-uniform) contribution of each signal modality to stress detection, we adopted several tree-based ensemble models: LightGBM, Random Forest, Gradient Boosting, and Extreme Gradient Boosting (XGBoost). These models are widely used in stress-detection research due to their strong predictive performance and interpretable feature contribution measures (for example, using SHAP values[1–3]; see **Supplementary Fig. 28D**). All models were trained using a leave-one-subject-out cross-validation (LOOCV) strategy to ensure a subject-independent evaluation. For each held-out subject, a model was trained and validated on the remaining subjects. Hyperparameters were optimized via grid search, using an inner loop where one subject's data served as validation and the rest as training data. For each model, we explored a range of hyperparameters and selected the configuration with the best validation performance. In the case of LightGBM, we tested number of trees in {200, 250, 300, 350, 400, 450, 500, 550, 600}, maximum tree depth in {1, 5, 10, 15}, and learning rate in {0.001, 0.01, 0.1, 1.0}, finding the best results with 200 trees, depth 5, and learning rate 0.1. For the Random Forest, we varied the number of estimators (trees) in {10, 40, 60, 80, 100, 150, 200}, maximum depth in {2, 4, 8, 10, 12, 14}, and minimum samples per split in {3, 5, 10, 15}; the optimal configuration was 200 trees, depth 4, and a minimum split size of 3. For XGBoost, we tried a number of estimators in {50, 100, 200, 500, 700}, learning rate in {0.001, 0.01, 0.1, 0.2}, and max depth in {2, 4, 8, 10, 15}, obtaining the best performance with 500 estimators, depth 8, and learning rate 0.1. Finally, for Gradient Boosting, the number of estimators was varied in {100, 200, 500, 700, 900, 1000}, learning rate in {0.001, 0.01, 0.1, 0.2}, and max depth in {2, 4, 8, 10, 12, 14}, with the best results at 200 estimators, depth 4, and learning rate 0.1. After hyperparameter tuning, we evaluated each ensemble model's performance (using the held-out subjects in LOOCV) on the binary stress classification task. LightGBM achieved an average accuracy of 75.11%, precision of 77.44%, recall of 75.11%, and F1-score of 74.59%. Random Forest performed better, with an average accuracy of 81.52%, precision of 82.65%, recall of 81.52%, and F1-score of 81.36%. Gradient Boosting yielded 83.17% accuracy, 84.85% precision, 83.17% recall, and 82.93% F1-score. XGBoost was the top performer among the models, with 84.42% average accuracy, 85.19% precision, 84.42% recall, and 84.33% F1-score. Because XGBoost slightly outperformed the others, we selected it for a detailed feature contribution analysis. We computed SHAP values from the trained XGBoost model to quantify each modality's relative importance in the stress predictions. The SHAP results revealed that feature contributions are indeed heterogeneous across modalities (**Supplementary Fig. 28D**). These findings support our motivation for using an attention-based fusion mechanism in Inception-MABFDNN: the model can learn to assign greater weight to the most informative signals during fusion. To further illustrate the importance of each modality for stress detection, as a model of study, we conducted an experiment using our DNN model. We retrained the Inception-MABFDNN after excluding the ECG signal, using only RAP, GSR, ST, and cortisol as inputs. The performance dropped from 93.35% accuracy (93.81% precision, 93.34% recall, 93.32% F1) when using all signals, down to 87.32% accuracy (87.63% precision, 86.91% recall, 87.32% F1) without ECG. This substantial decrease in accuracy confirms the distinct contribution of ECG-derived patterns to stress detection in our multimodal framework. It also underscores that all modalities add value; the information contained in each signal is uniquely important.

XGBoost vs. Inception-MABFDNN: Performance Comparison. To bridge the interpretable classical approach with our deep learning approach, we directly compared XGBoost and Inception-MABFDNN under the same subject-independent LOOCV evaluation. XGBoost achieved 84.42% accuracy, whereas Inception-MABFDNN achieved 93.35% accuracy on the binary stress classification task. This performance gap demonstrates the superior capacity of our deep model to learn hierarchical, multimodal representations of the stress response. Inception-MABFDNN was better able to integrate the complementary signals and capture complex patterns, yielding significantly higher accuracy in distinguishing stress from non-stress states.

Supplementary Information Tables:
Supplementary Table. 1 | Glossary of frequently used abbreviations.

Abbreviations	Full Form
SQC-SAS	Smart Quantitative and Comprehensive Stress Assessor and Sub-Classifier
MSB	Molecular Stress Biomarkers
PSI	Physiological Stress Indicators
ML	Machine Learning
Inception-MABFDNN	Inception Multimodal Attention-Based Fusion Deep Neural Network
1D-CNN	One-Dimensional Convolutional Neural Networks
WLR	Wireless Reader
BLOSDA	Battery-Less On-Skin Data Acquisition System
ECG	Electrocardiograms
GSR	Galvanic Skin Response
RAP	Radial Artery Pulse
ST	Skin Temperature
IDE	Interdigitated Electrodes
IP	Iontophoresis
MIP	Molecularly Imprinted Polymer
NIP	Non-Imprinted Polymer
M&M	Multimodal And Multi-Sensing
NFC	Near-Field Communication
MCU	Microcontroller Unit
PBS	Phosphate-Buffered Solution
ECA	Electrochemical
DPV	Differential Pulse Voltammetry
LSV	Linear Sweep Voltammetry

Supplementary Table. 2 | Key stress-related features across different physiological signals as identified in previous studies.

ECG	Description	Reference
HR	Average number of R-peaks per minute	
SDRR	Standard deviation of the time intervals between successive RR intervals	
pRR50	Percentage of adjacent RR intervals that differ by more than 50 milliseconds	[54,55]
MinRR	Shortest recorded RR interval	
MaxRR	Longest recorded RR interval	
TP	Sum of powers of all the frequency components in RR intervals variation (0-0.4 Hz)	
**RAP**	**Description**	**Reference**
T_{pi}	Pulse interval, time between the pulse onset and pulse offset	
CT	The time between the pulse onset and systolic peak	
A_{sp}	Systolic peak amplitude, the difference in amplitude between the pulse onset and systolic peak	
A_{dn}	Dicrotic notch amplitude, the difference in amplitude between the pulse onset and dicrotic notch	[44,56]
A_{dp}	Diastolic peak amplitude, the difference in amplitude between the pulse onset and diastolic peak	
IPR	Instantaneous pulse rate	
**GSR**	**Description**	**Reference**
SCR Peaks	The number of significant rises in skin conductance response	
Peaks Amp.	The magnitude of the SCR peaks	
SCR RiseT	Duration from SCR onset to peak	[57]
SCR RecT	Time from the peak back to baseline, showing recovery duration	
SCR Mean	Average SCR level	
SCR STD	Variability of SCR amplitudes around the mean level	
**ST**	**Description**	**Reference**
ST Mean	The average of ST	
ST STD	Variability of ST around the mean level	[58]
ST PSD Mean	Average power across the ST frequency spectrum	
ST PSD STD	Average power across the ST frequency spectrum	

Supplementary Table. 3 | Comparison of commercial wearables and our device in stress monitoring features, including PSI/MSB sensing, simultaneous monitoring, stress detection, subtype analysis, and their accuracies.

Devices	Commercial Watch 1	Commercial Watch 2	Ours
**PSI measurements**	ECG, PPG, ST	PPG	ECG, GSR, RAP, ST
**MSB measurements**	No	No	Cortisol
**Simultaneous monitoring**	No	N/A	Yes
**Stress detection**	Yes	Yes	Yes
**Stress subtype detection**	No	No	Yes
**False positive/False negative accuracy (%) during physical activity–light exercise (n=3)**	5	0	95
**False positive/False negative accuracy (%) during standing (n=3)**	25	0	100
**False positive/False negative accuracy (%) during Environmental stress (CPT) (n=3)**	49	91	94
**False positive/False negative accuracy (%) during Cognitive stress (Math Quiz) (n=3)**	67	89	91
**Signal used for stress detection**	PPG only	PPG only	Cortisol, ECG, GSR, RAP, ST

Supplementary Table. 4 | Comparison of existing wearable biosensing systems and our developed device for stress assessment. Features compared include sensed physical and molecular signals, sweat collection methods, sensor reusability, device flexibility, power source, machine learning capability, and stress assessment methodology.

Devices	Physical Signals	Molecular Signals	In-sensor Restoration Capability	Automatic On-demand Sweat Generation	Long-term Wearable and Environmental Stability	Sweat Sample Refreshing Capability	Continues, Long-term, while Non-invasive Sweat Sampling	Sensor Flexibility	Readout Circuit Flexibility	Power Source	Low Power Wireless Data Energy Exchange	Machine Learning	Stress Assessment Methodology
59	No	Sweat cortisol	No	No	Yes	No	No	Flexible	Flexible	Wired battery	No	No	Cortisol concentration analysis
62	GSR	No	N/A	N/A	N/A	N/A	N/A	Flexible	Non-flexible	Wired battery	No	No	Statistical analysis of GSR
63	GSR, ST	No	N/A	N/A	N/A	N/A	N/A	Flexible	Flexible	Wired battery	No	No	Statistical analysis of GSR
64	PPG	Sweat cortisol	No	No	No	No	No	Flexible + non-flexible	Non-flexible	Wired battery	No	No	Statistical analysis of HRV and cortisol concentration
65	ST	Saliva or sweat cortisol	No	No	No	No	No	Flexible	Flexible	Wired battery	No	No	Cortisol concentration analysis
**Ours**	ECG, RAP, GSR, ST	Sweat cortisol	Yes	Yes	Yes	Yes	Yes	Flexible	Flexible	Wireless powered via NFC	Yes	Multimodal Attention-Based Fusion Deep Neural Network	Classification of various types of stress in daily life

Supplementary Information Figures:

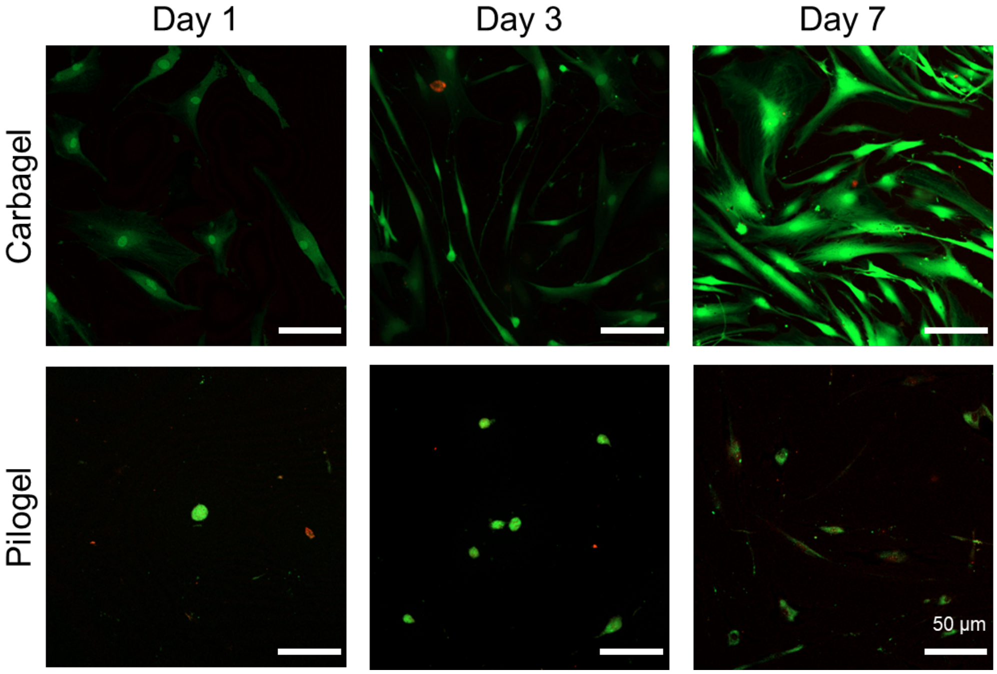

Supplementary Fig. 1 | Biocompatibility Assessment of IP gels with Fibroblast Cells. (A) Image representatives of live/dead fluorescent micrographs of normal human dermal fibroblast cells cultured at 1, 3, and 7 days in direct contact with Carbagel and Pilogel (scale bar: 50 µm).

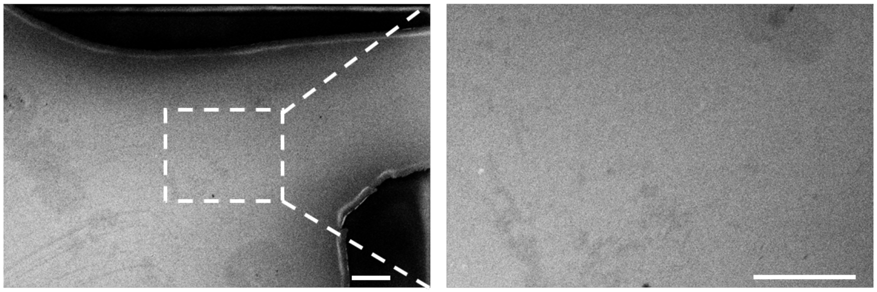

Supplementary Fig. 2 | Mechanical stability assessment of the SQC-SAS wearable's PSI patch: bending and stretching analyses. SEM images of the PSI patch electrodes (Au/PI film) surface after 10,000 cycles of 30% bending and 2,000 cycles of 20% stretching, showing no defects or cracks (scale bar: 100 μm).

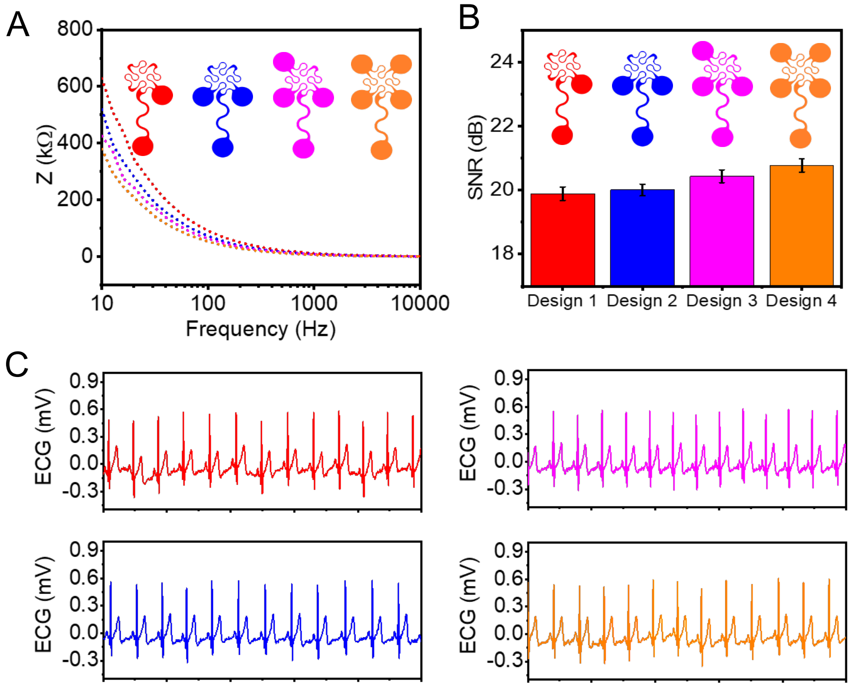

Supplementary Fig. 3 | Skin-electrode interface impedance study and SNR analysis of electrodes with various designs. (A-C) Study on the effect of the number of ECG electrode islands on (A) electrode-skin contact impedance, (C) real-time ECG signals collected, and (B) the signal-to-noise ratio (SNR) of the ECG signals (n = 3 technical replicates). Data are presented as mean values + /− SD.

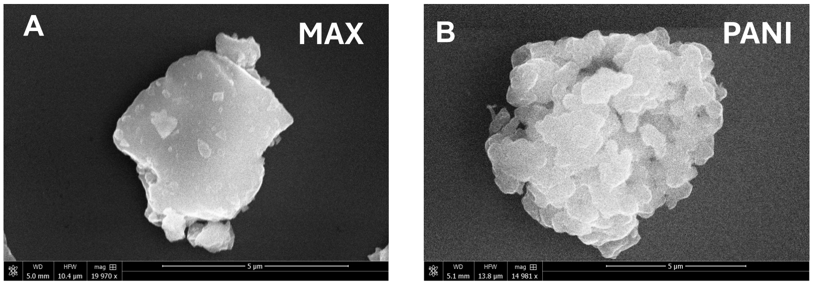

Supplementary Fig. 4 | SEM images of RAP sensor's e-textile composite. SEM image of **(A)** MAX and **(B)** PANI.

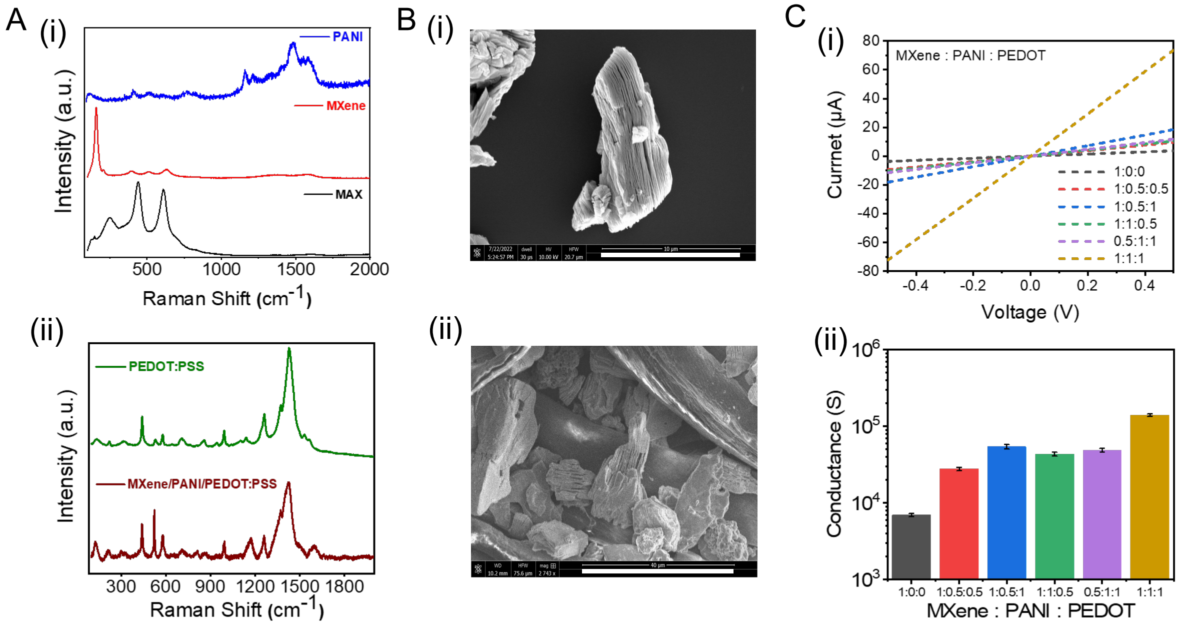

Supplementary Fig. 5 | Material and Electrical characterization of the RAP sensor's conductive e-textile. (A) Raman spectra of (i) PANI, MXene and MAX phase and (ii) PEDOT and MXene/PANI/PEDOT composites. (B) SEM image of (i) MXene (Scale bar: 10 μm) and (ii) MXene/PANI/PEDOT coated e-textile composite (Scale bar: 40 μm) for the RAP sensor. (C) Current-voltage characteristic curves of e-textiles (i) and (ii) conductance of various MXene/PANI/PEDOT ratios (n = 3 technical replicates). Data are presented as mean values + /− SD.

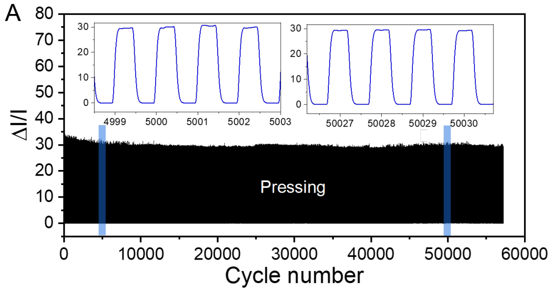

Supplementary Fig. 6 | Mechanical stability of RAP sensor. Mechanical stability tests conducted on the RAP sensor under **(A)** pressing with a pressure of 10 kPa and **(B)** 30% bending for over 50,000 cycles.

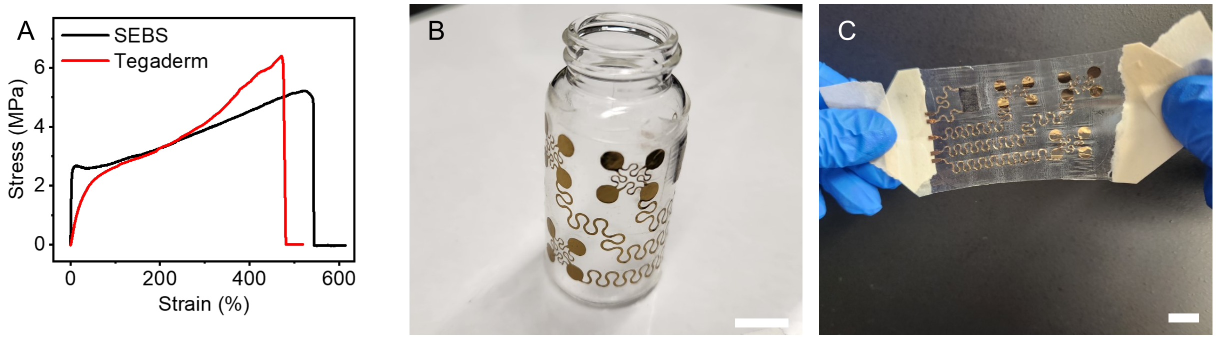

Supplementary Fig. 7 | Evaluation of M&M Panel's Skin Conformability and Mechanical Integrity. (A) Comparison of the stress-strain curves for the SEBS substrate and Tegaderm film. **(B)** Flexibility of the M&M panel under bending. **(C)** Stretchability of the M&M panel under stretching. (Scale bar: 1 cm)

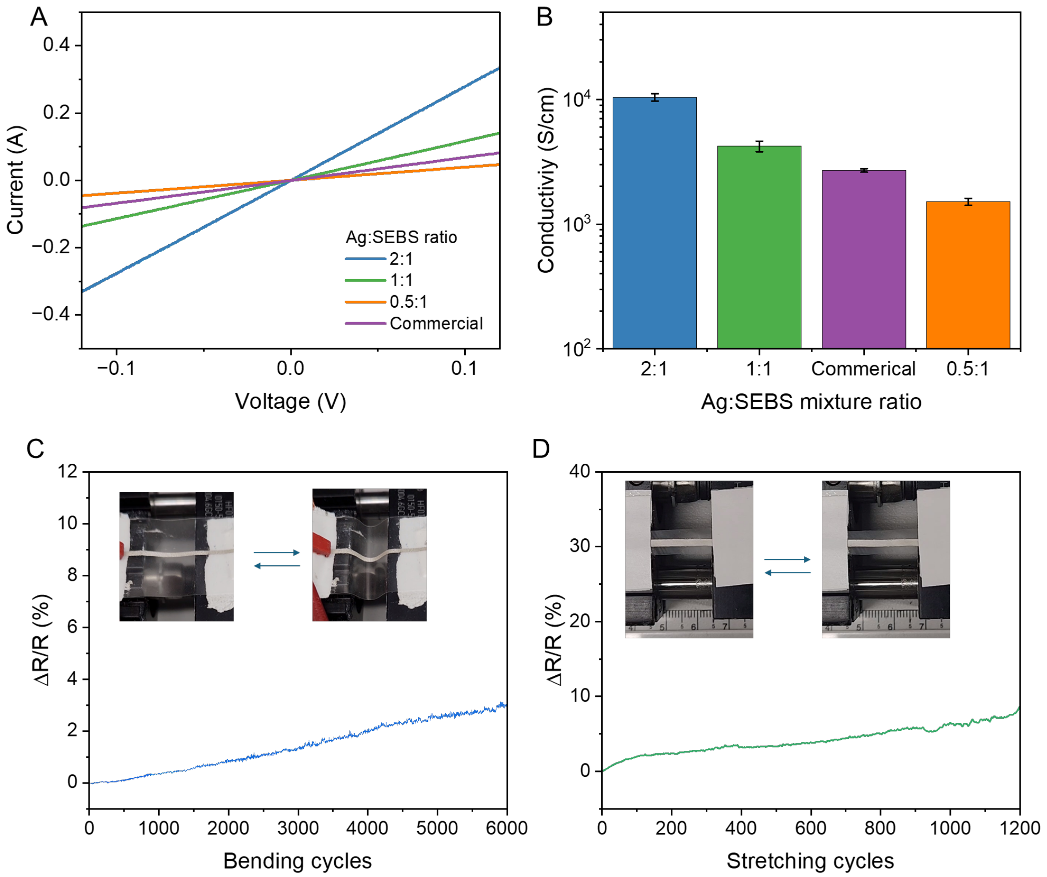

Supplementary Fig. 8 | Evaluation of conductivity and mechanical stability of the conductive Ag/SEBS ink. (A) I-V curves of the Ag/SEBS ink with various ratios compared with a commercial Ag/AgCl ink **(B)** Conductivity of the Ag/SEBS ink with various ratios compared with a commercial Ag/AgCl ink (n = 3 technical replicates). Data are presented as mean values + /− SD. **(C)** Relative resistance changes of the conductive trace printed with Ag/SEBS ink measured after repeated 30% bending cycles, up to a total of 6000 cycles. **(D)** Relative resistance changes of the conductive trace printed with Ag/SEBS ink measured after repeated 20% stretching cycles, up to a total of 1200 cycles.

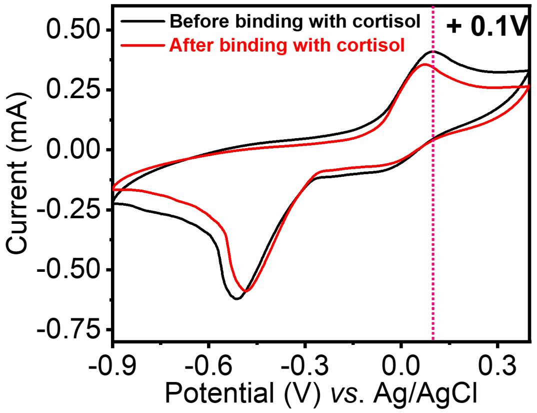

Supplementary Fig. 9 | Electrochemical validation of MIP preparation on SQC-SAS wearable's MSB electrode arrays. CV of the MIP electrode before and after its 2-minute binding with the existence of cortisol biomolecule in 0.1 M PBS. (Scan rate: 0.05 V/s, Cortisol concentration: 10 μM).

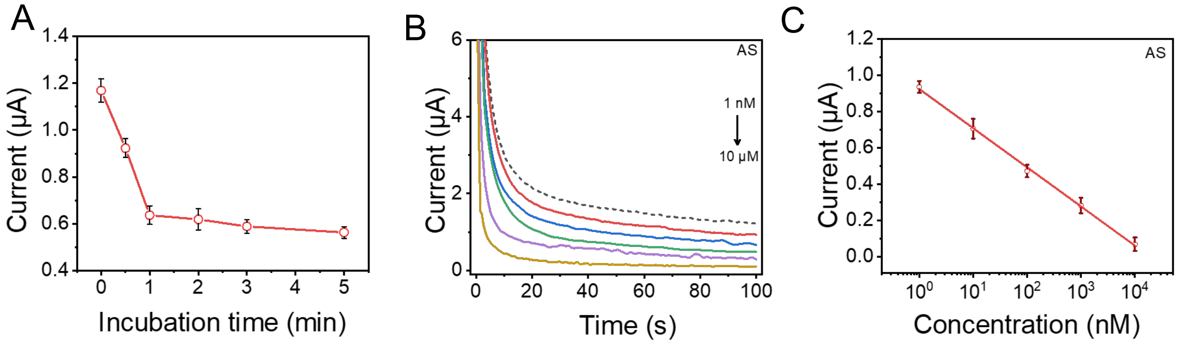

Supplementary Fig. 10 | Characterization of the SQC-SAS wearable's MSB patch: Incubation, and repeatability. (A) Optimization of incubation time for the interaction of cortisol biomolecules with their specific MIP matrix (n = 3 technical replicates). Data are presented as mean values +/− SD. **(B)** ECA response of the MIP cortisol sensor to different cortisol concentrations (1 nM- 10 µM) in AS. **(C)** Corresponding calibration plots of the MIP cortisol sensors in AS. MIP sensor shows a logarithmic response of the electrode current to the cortisol level (n = 3 technical replicates). Data are presented as mean values + /− SD.

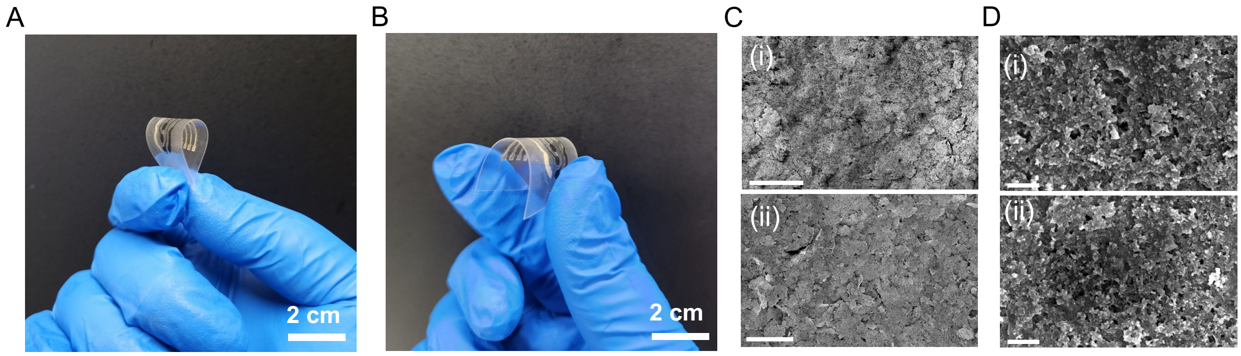

Supplementary Fig. 11 | Investigating the mechanical stability of SQC-SAS wearable's MSB patch. (A-B) Optical images showing the flexibility of the MSB sensor under bending conditions. **(C)** Ag reference electrode (i) before and (ii) after 1000 bending cycles (scale bar: 50 μm) and **(D)** MIP working electrode (i) before and (ii) after the 1000 bending cycles (scale bar: 1 μm).

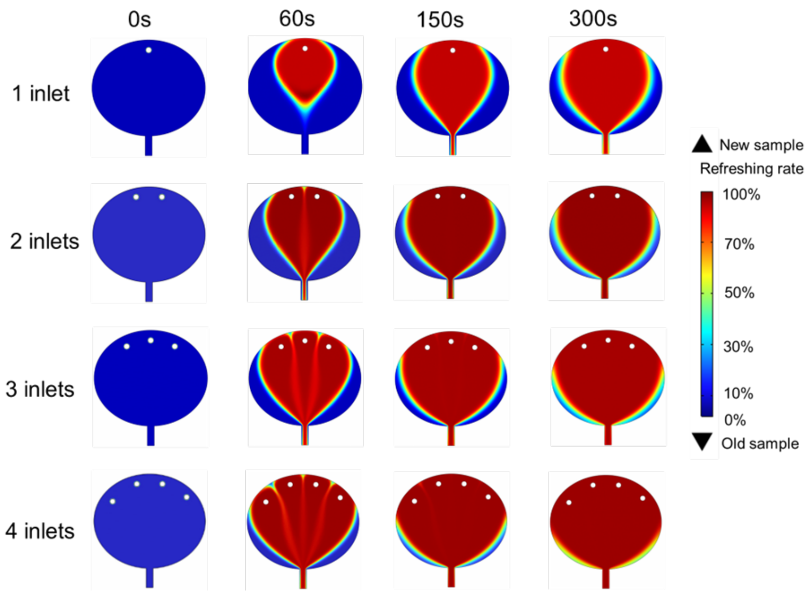

Supplementary Fig. 12 | Simulation of the effect of microfluidic inlet number on channel refreshing rate. Simulations results for refreshing efficiency of microfluidic channels with various inlet design

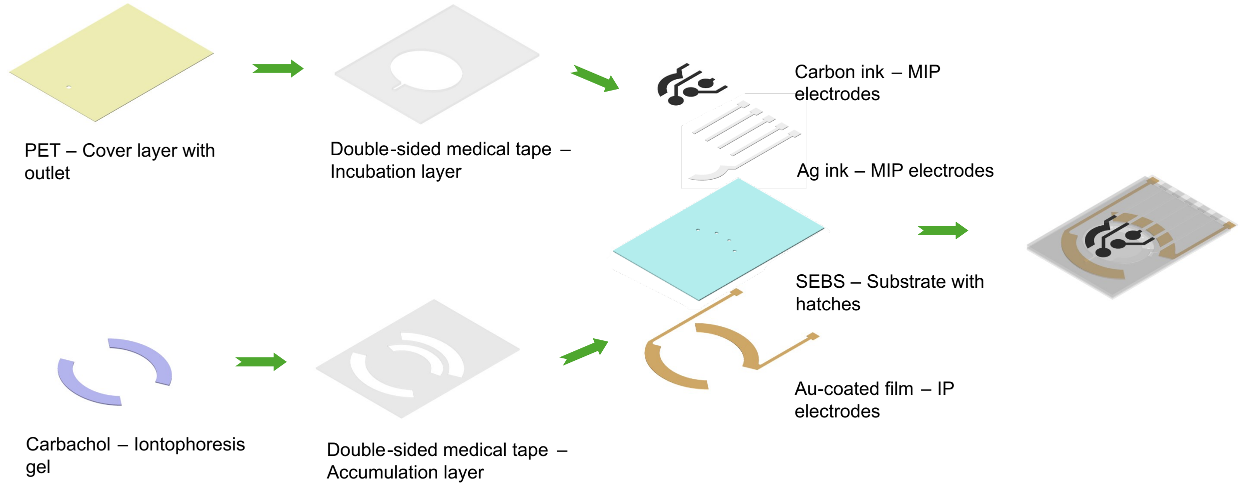

Supplementary Fig. 13 | Schematic Illustration of the MSB Fabrication Process. Schematic representation of the MSB step-by-step fabrication process.

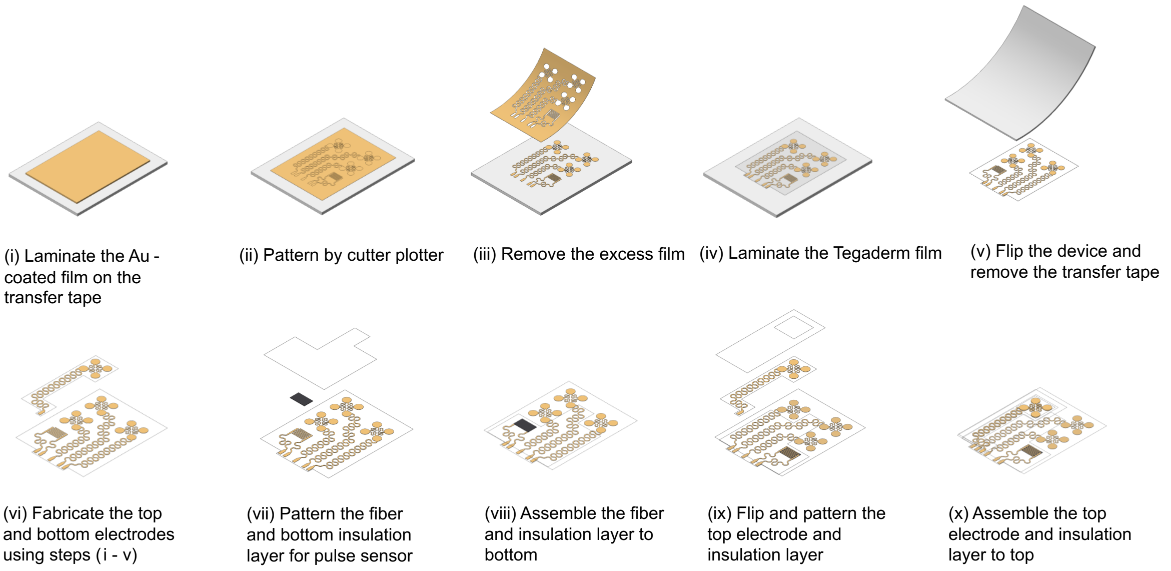

Supplementary Fig. 14 | Schematic Illustration of the PSI patch Fabrication Process. Schematic representation of the ECG and GSR electrodes and RAP sensor step-by-step fabrication process.

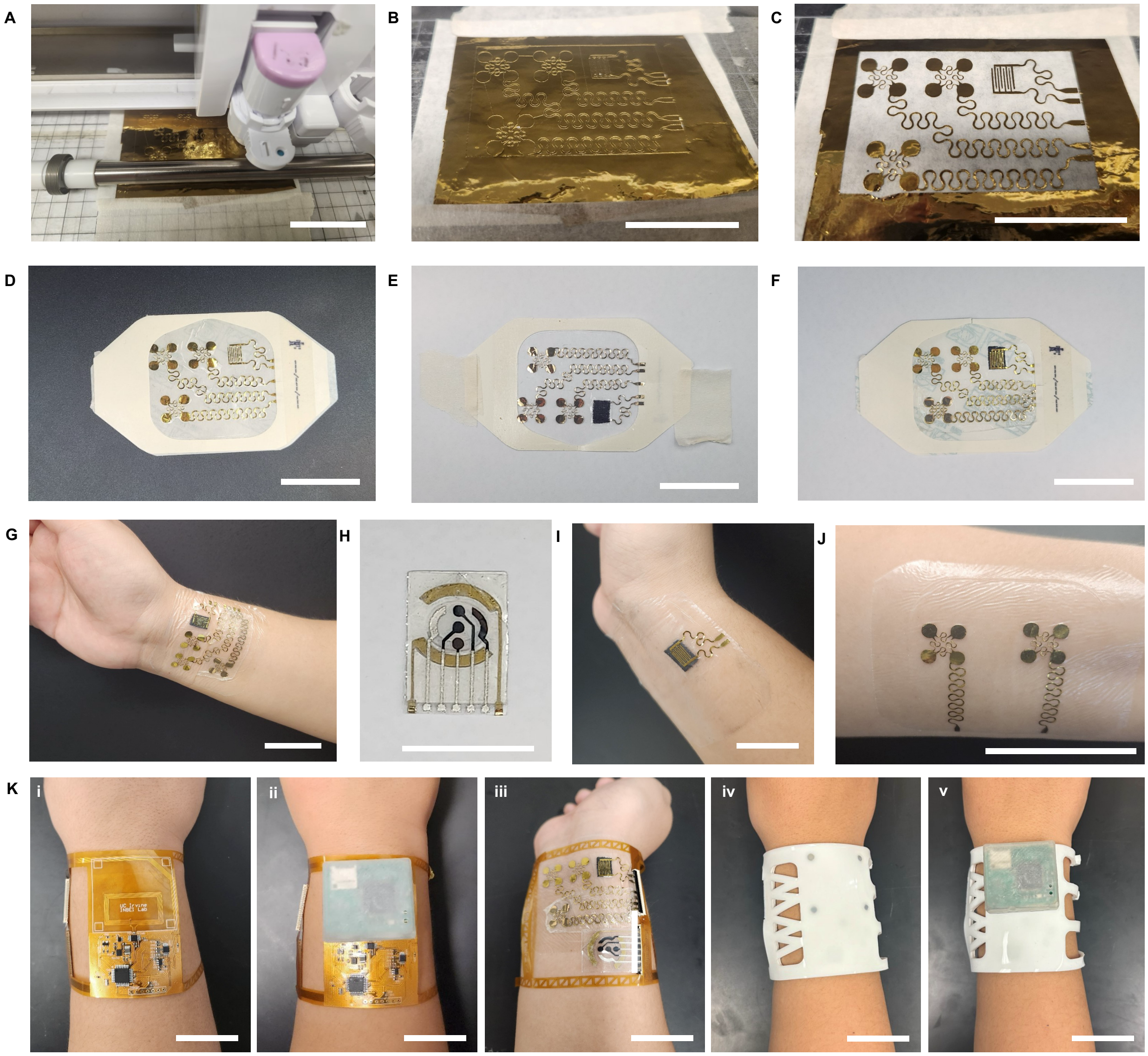

Supplementary Fig. 15 | Optical photos of the SQC-SAS wearable Fabrication Process. Step-by-step M&M Fabrication Process and device assembly. (A) Patterning the Au/PI film using cutter plotter. Patterned PSI patch **(B)** before and **(C)** after removing excess films. **(D)** Patterned PSI patch transferred to Tegaderm film. **(E)** Assembled PSI patch with bottom-side layers. **(F)** Fully assembled PSI patch with top-side layers **(G)** PSI patch attached to wrist **(H)** Fully assembled MSB patch. **(I)** Individual RAP sensor and **(J)** ECG or GSR sensors attached to wrist. **(K)** (i) BLOSDA FPCB attached to the outer wrist. (ii) SQC-SAS wearable showing BLOSDA FPCB and WLR attached to the outer wrist. (iii) M&M panel connected to the BLOSDA FPCB and attached to inner wrist. (iv) Top side of the BLOSDA FPCB with silicone encapsulation. (v)SQC-SAS wearable showing silicone encapsulated BLOSDA FPCB and WLR attached to outer wrist. (scale bar: 3 cm).

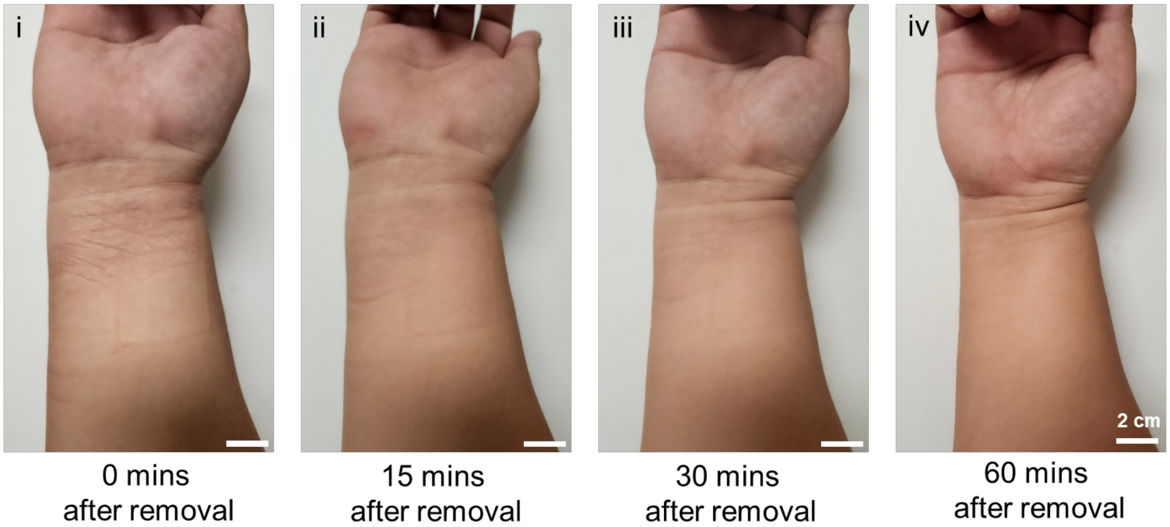

Supplementary Fig. 16 | On-Body Biocompatibility Assessment of SQC-SAS wearable's M&M Panel. Photographic illustrations demonstrating the on-body biocompatibility of the M&M panel. The subject wore the device for 8 hours, with figure sets showing the visual effects on the skin immediately after the removal of the sensor array (0 minutes) and up to 60 minutes post-removal (scale bar: 2 cm). The skin recovered to normal within 1 hour after removal, with no allergic reactions observed.

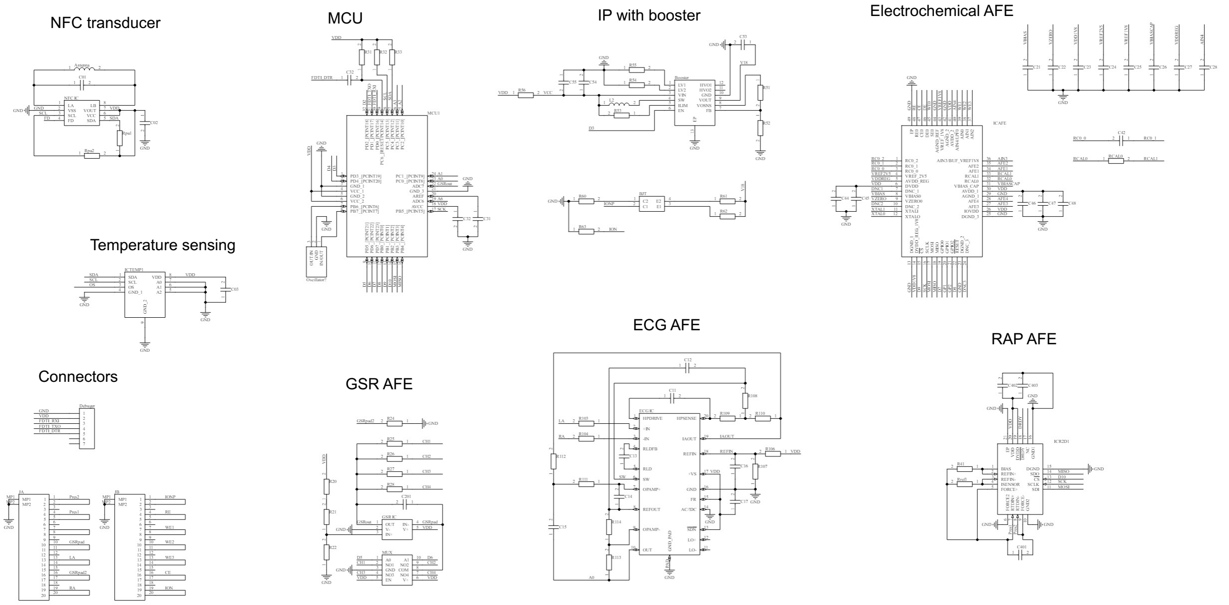

Supplementary Fig. 17 | Detailed circuit schematic of the BLOSDA for the advanced SQC-SAS wearable.

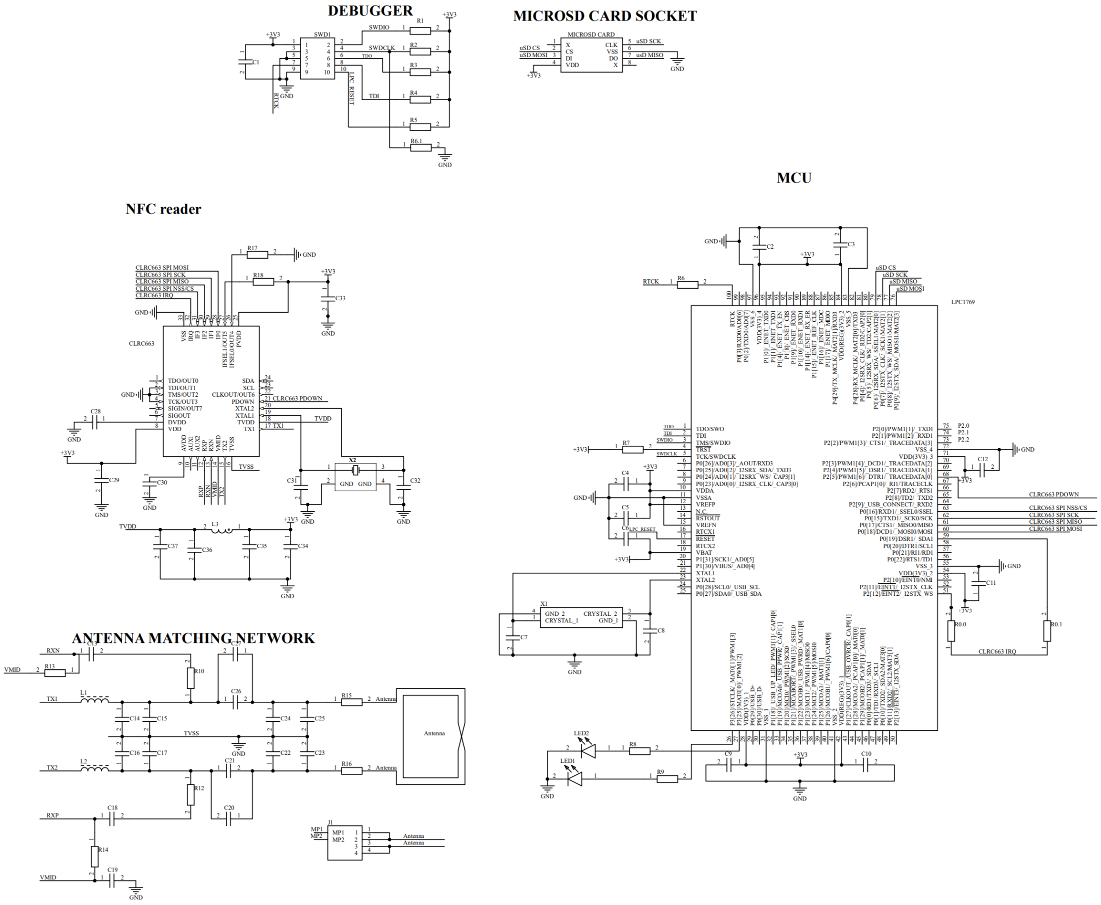

Supplementary Fig. 18 | Detailed circuit schematic of the WLR for the advanced SQC-SAS wearable.

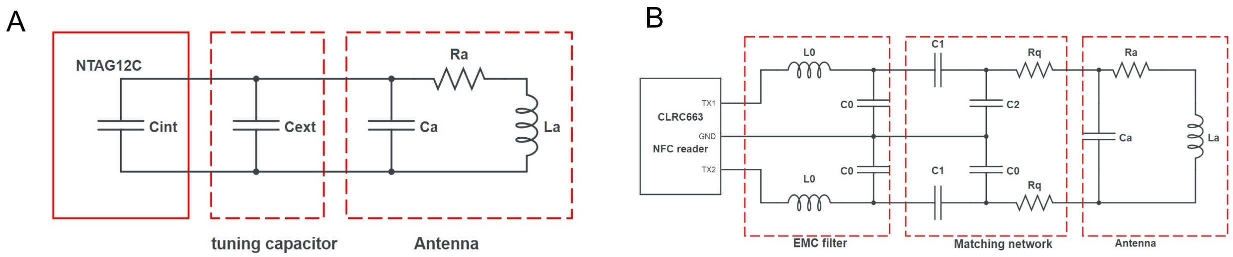

Supplementary Fig. 19 | NFC antenna characterization. NFC front-end equivalent circuit of (A) tag (BLOSDA) and **(B)** reader (WLR).

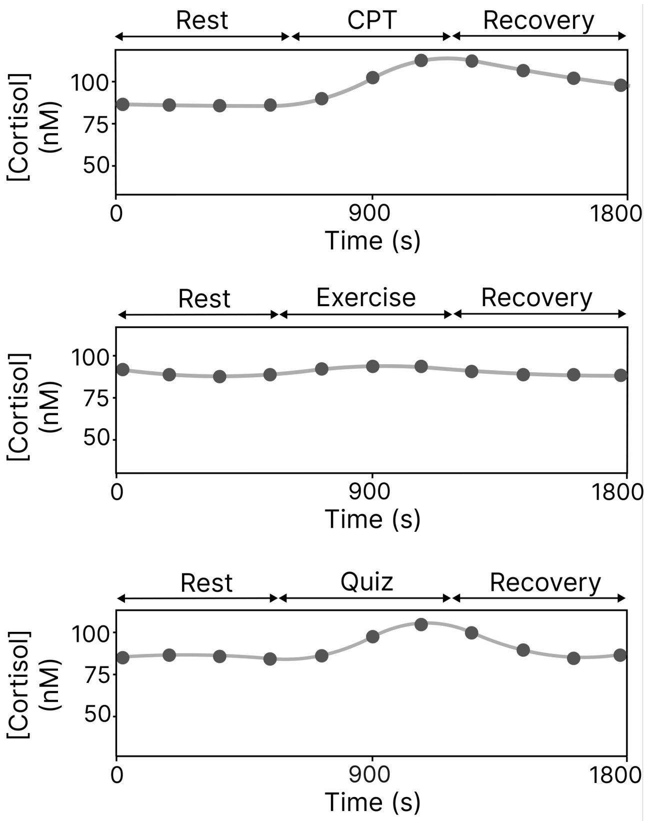

Supplementary Fig. 20 | Cortisol concentration measurements in response to the CPT, exercise, and a mathematical quiz.

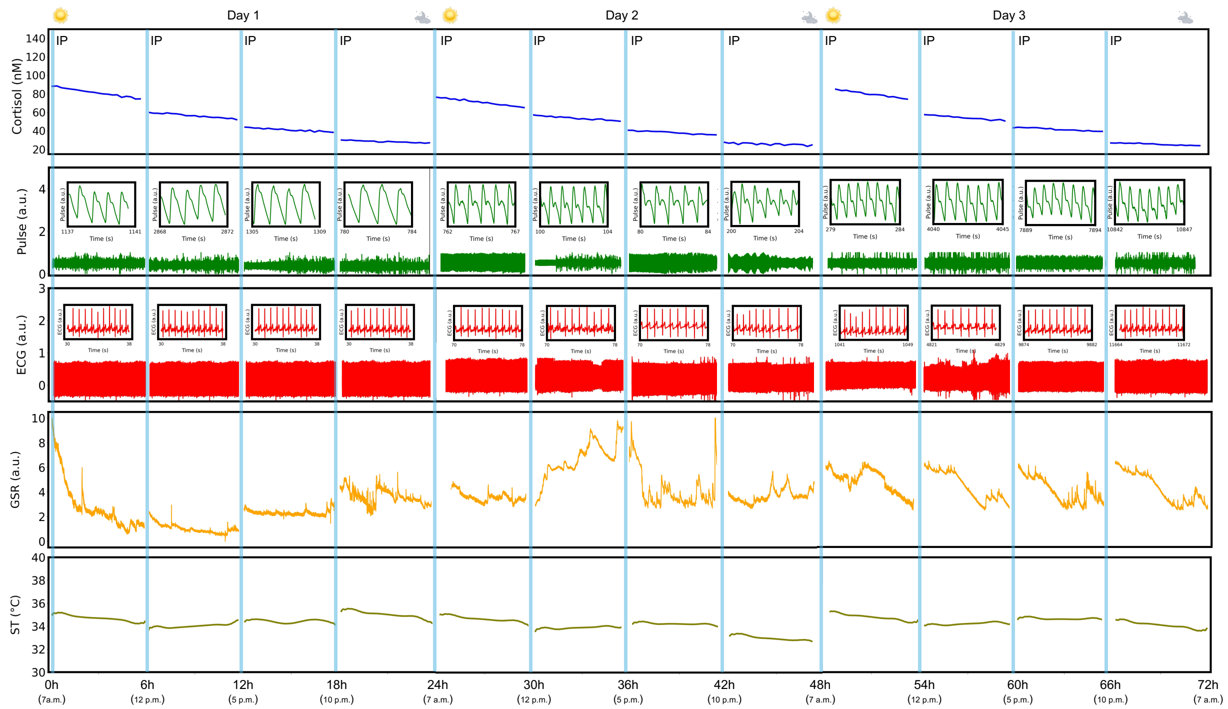

Supplementary Fig. 21 | Prolonged wireless and multimodal stress monitoring over 72 hours (3 days). Demonstration of the SQC-SAS wearable system's prolonged functionality, capturing molecular stress biomarkers and physiological stress markers over a 72-hour period. Extracted features include cortisol concentration, RAP, ECG, GSR, and ST, underscoring the device's stability and robustness for extended real-world stress assessment.

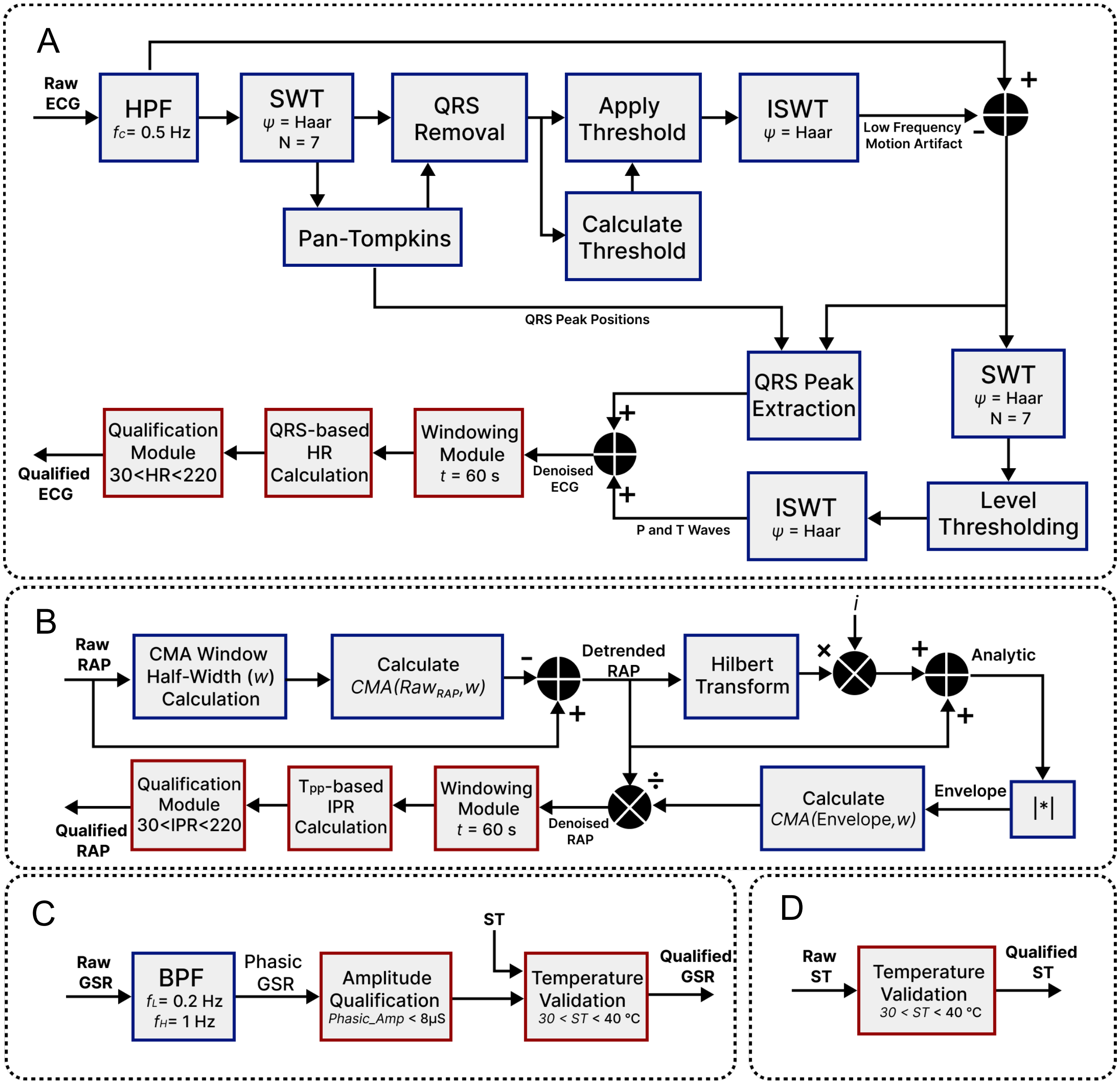

Supplementary Fig. 22 | Overview of physiological signals qualification and denoising workflows for (A) ECG, (B) RAP, (C) GSR, and (D) ST. Each workflow includes steps for noise reduction and signal qualification.

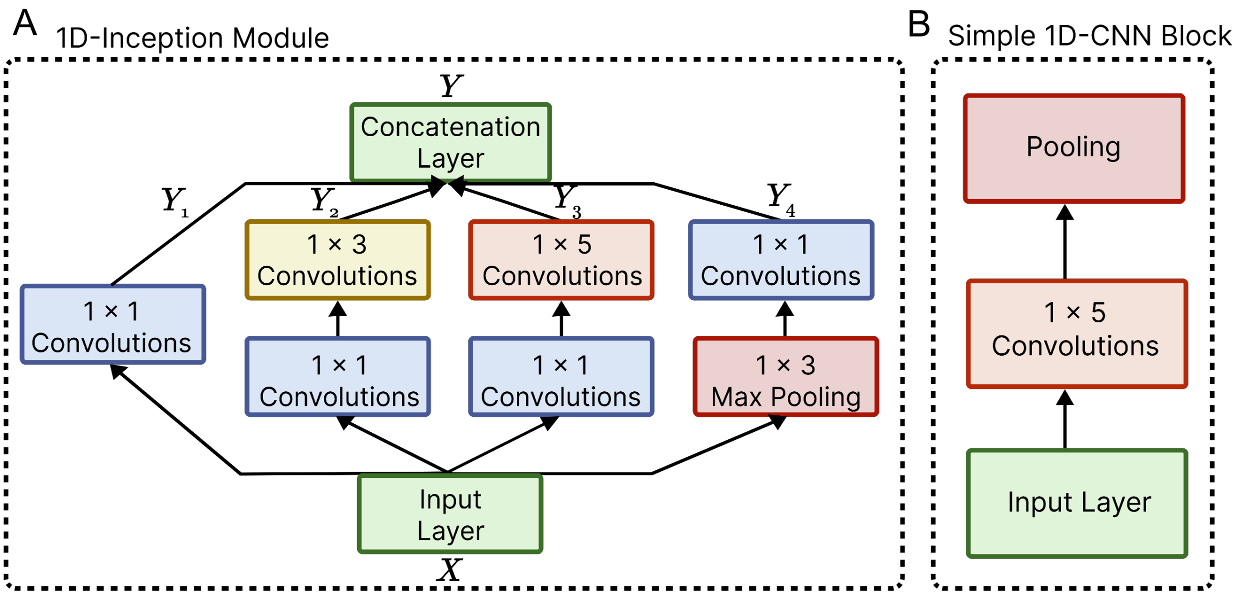

Supplementary Fig. 23 | Overview of 1D-CNN and 1D-Inception Module structures. (A) 1D-Inception Module with different filter sizes adapted for 1D signal analysis. (B) A simple 1D-CNN layer structure.

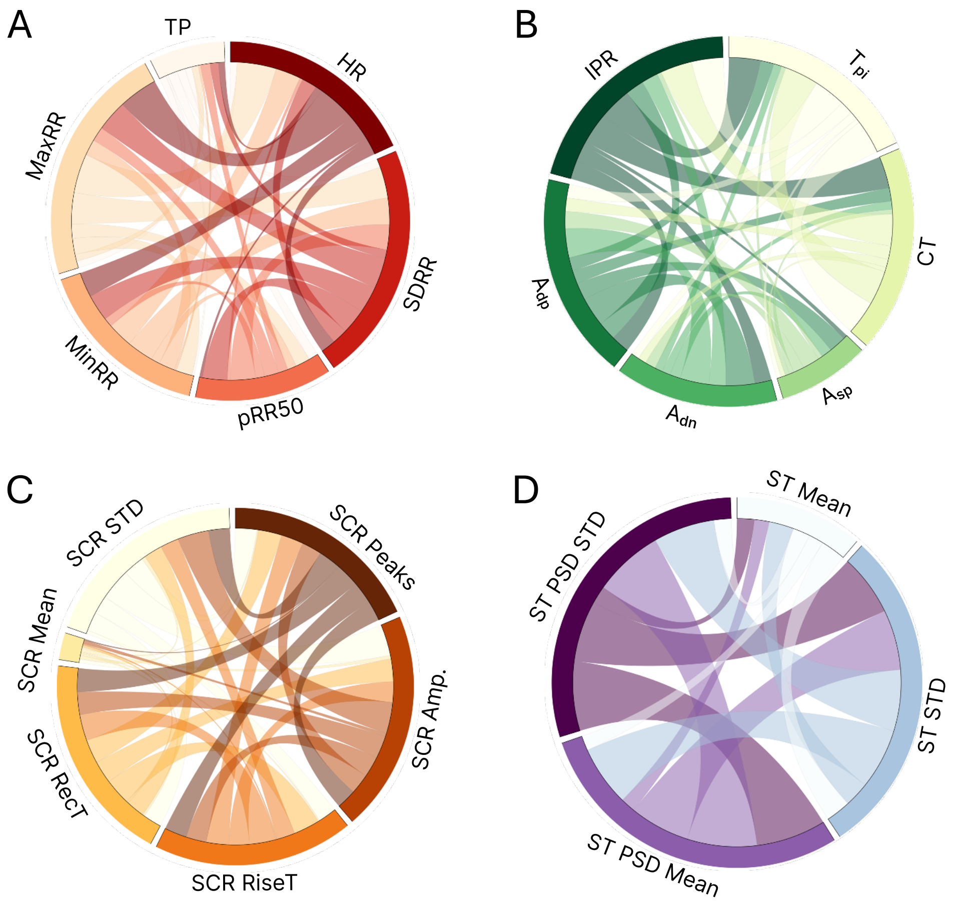

Supplementary Fig. 24 | Chord diagrams that display the correlation relationships of features specific to each modality. (A) ECG, (B) RAP, (C) GSR, and (D) Skin Temperature.

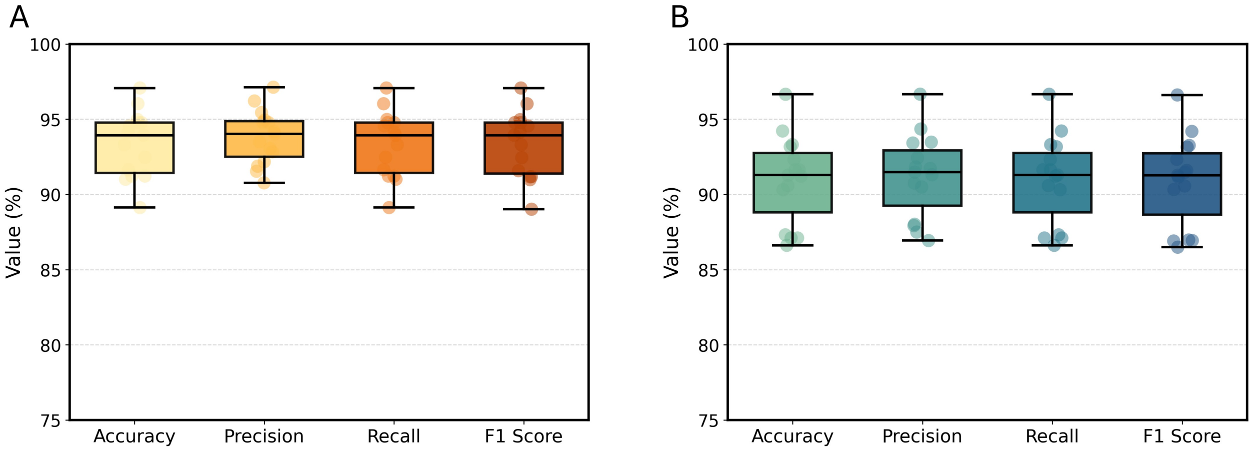

Supplementary Fig. 25 | Subject-independent classification performance. (A) Classification of "Stress" vs "Rest" states and (B) Subclassification of stress types. Leave-One-Out Cross-Validation (LOOCV) was applied at the subject level to evaluate model generalizability across individuals. In each iteration, data from one subject was reserved for testing, while the remaining subjects' data were used for training. (center line, median; box limits, upper and lower quartiles; whiskers, 1.5x interquartile range)

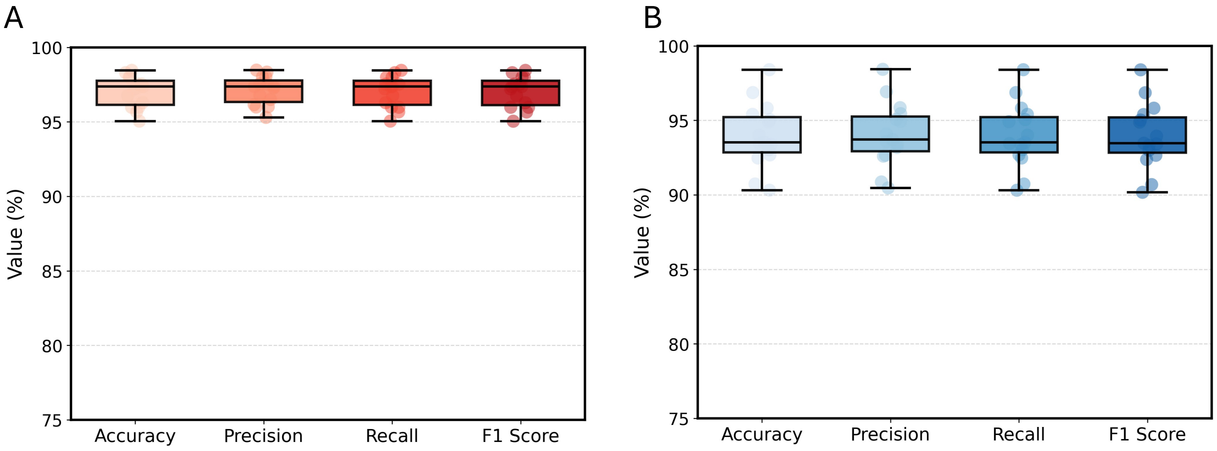

Supplementary Fig. 26 | Subject-dependent classification performance with session-level fine-tuning. (A) Classification of "Stress" vs "Rest" states and (B) Subclassification of stress types. For each subject, the model was initially trained on data from all other subjects (excluding the target subject) and then fine-tuned by updating only the decision layer using data from one session of the target subject. The fine-tuned model was evaluated on data from the remaining session of the same subject. (center line, median; box limits, upper and lower quartiles; whiskers, 1.5x interquartile range)

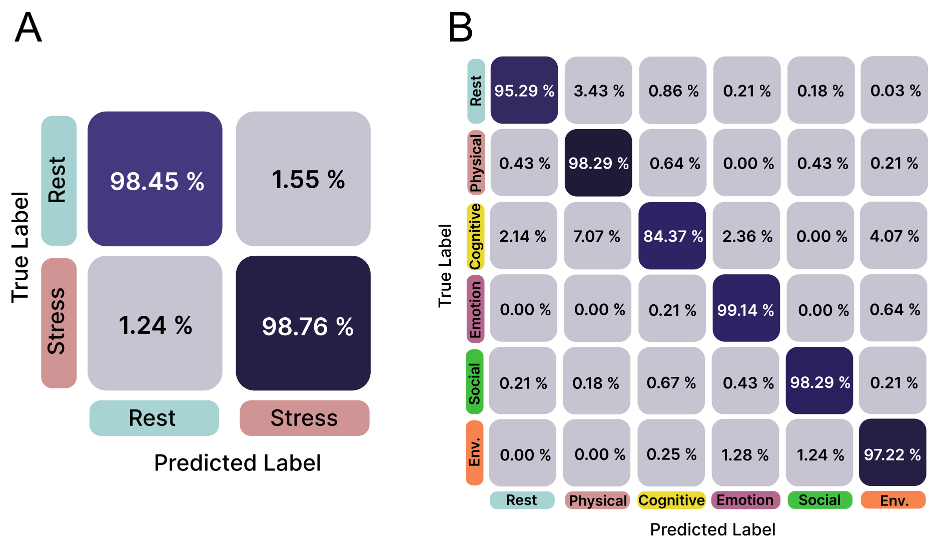

Supplementary Fig. 27 | Representative confusion matrices for subject-dependent evaluation. (A) Classification of "Stress" vs Not Stress (Labeled as "Rest") states, and (B) subclassification of stress types.

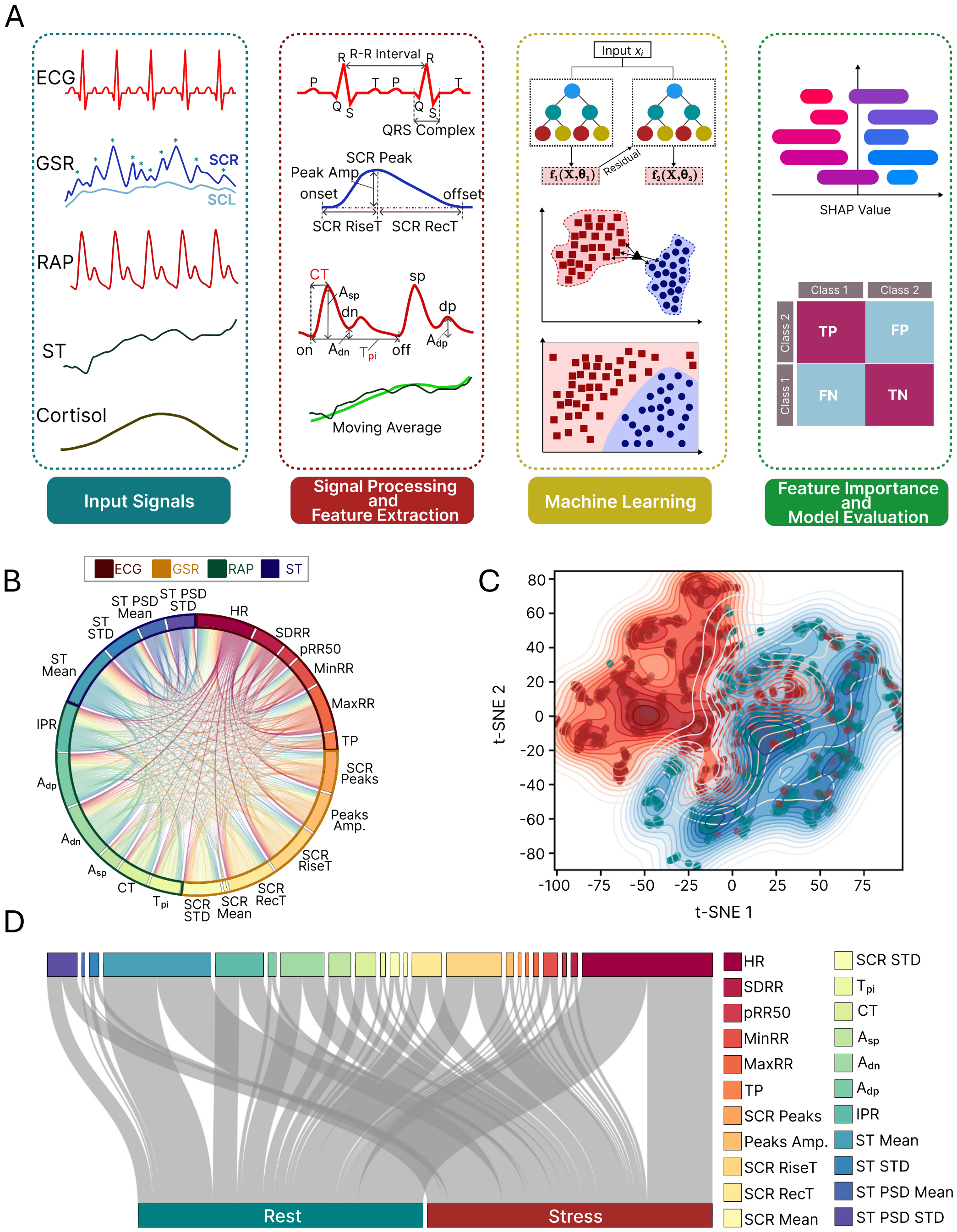

Supplementary Fig. 28 | The explanatory analysis steps and results for dataset characterization, interpretability, and validation of Inception-MABFDNN design. **(A)** Four stages of our analysis: (1) signal collection, (2) preprocessing and feature extraction, (3) using interpretable ML algorithms, and (4) SHAP values analysis and performance evaluation. **(B)** Representative Chord diagram showing interrelationships among stress-related features using PCC. **(C)** t-SNE projection of feature space into 2D clusters for stress and rest states. **(D)** Representative SHAP values indicating feature importance using the XGBoost classifier.

Supplementary References

1 Lee, S. W. *et al.* Graphene-based electronic textile sheet for highly sensitive detection of NO2 and NH3. *Sensors and Actuators B: Chemical* **345**, 130361 (2021).

2 Lee, S. W. *et al.* Bio-inspired electronic textile yarn-based NO2 sensor using amyloid–graphene composite. *ACS sensors* **6**, 777-785 (2020).

3 Zheng, Y. *et al.* Conductive MXene/cotton fabric based pressure sensor with both high sensitivity and wide sensing range for human motion detection and E-skin. *Chemical Engineering Journal* **420**, 127720 (2021).

4 Zhao, X. *et al.* Smart Ti3C2T x MXene fabric with fast humidity response and joule heating for healthcare and medical therapy applications. *ACS nano* **14**, 8793-8805 (2020).

5 Liu, X. *et al.* Air-permeable, multifunctional, dual-energy-driven MXene-decorated polymeric textile-based wearable heaters with exceptional electrothermal and photothermal conversion performance. *Journal of Materials Chemistry A* **8**, 12526-12537 (2020).

6 Sharma, S., Chhetry, A., Sharifuzzaman, M., Yoon, H. & Park, J. Y. Wearable capacitive pressure sensor based on MXene composite nanofibrous scaffolds for reliable human physiological signal acquisition. *ACS applied materials & interfaces* **12**, 22212-22224 (2020).

7 Zhang, S. *et al.* 3D MXene/PEDOT: PSS composite aerogel with a controllable patterning property for highly sensitive wearable physical monitoring and robotic tactile sensing. *ACS Applied Materials & Interfaces* **14**, 23877-23887 (2022).

8 Zhu, Y. *et al.* Highly sensitive and skin-like pressure sensor based on asymmetric double-layered structures of reduced graphite oxide. *Sensors and Actuators B: Chemical* **255**, 1262-1267 (2018).

9 Rajendran, J. *et al.* Preparation of 2D Graphene/MXene nanocomposite for the electrochemical determination of hazardous bisphenol A in plastic products. *Chemosphere* **287**, 132106 (2022).

10 Rajendran, J. *et al.* 2D MXene/graphene nanocomposite preparation and its electrochemical performance towards the identification of nicotine level in human saliva. *Journal of Hazardous Materials* **440**, 129705 (2022).

11 Presser, V. *et al.* First-order Raman scattering of the MAX phases: Ti2AlN, Ti2AlC0. 5N0. 5, Ti2AlC,(Ti0. 5V0. 5) 2AlC, V2AlC, Ti3AlC2, and Ti3GeC2. *Journal of Raman Spectroscopy* **43**, 168-172 (2012).

12 Naguib, M. *et al.* in *MXenes* 15-29 (Jenny Stanford Publishing, 2011).

13 Rajendran, J. Amperometric determination of salivary thiocyanate using electrochemically fabricated poly (3, 4-ethylenedioxythiophene)/MXene hybrid film. *Journal of Hazardous Materials* **449**, 130979 (2023).

14 Cao, Y. *et al.* Enhanced thermal properties of poly (vinylidene fluoride) composites with ultrathin nanosheets of MXene. *RSC advances* **7**, 20494-20501 (2017).

15 Lee, S. W., Pei, X., Rajendran, J. & Esfandyarpour, R. A Wireless and Battery-Free Wearable Pressure Sensing System for Human-Machine Interaction and Health Monitoring. *IEEE Journal on Flexible Electronics* (2023).

16 Jeong, J. M. *et al.* Hierarchical hollow spheres of Fe 2 O 3@ polyaniline for lithium ion battery anodes. (2013).

17 Kumar, N. A. *et al.* Polyaniline-grafted reduced graphene oxide for efficient electrochemical supercapacitors. *ACS nano* **6**, 1715-1723 (2012).

18 Aeiyach, S. *et al.* (ACS Publications, 2003).

19 Cho, M., Yun, Y., Nam, J., Son, Y. & Lee, Y. Effect of magnetic field on electrochemical polymerization of EDOT. *Synthetic Metals* **158**, 1043-1046 (2008).

20 Garreau, S., Louarn, G., Buisson, J., Froyer, G. & Lefrant, S. In situ spectroelectrochemical Raman studies of poly (3, 4-ethylenedioxythiophene)(PEDT). *Macromolecules* **32**, 6807-6812 (1999).

21 Tran-Van, F., Garreau, S., Louarn, G., Froyer, G. & Chevrot, C. Fully undoped and soluble oligo (3, 4-ethylenedioxythiophene) s: spectroscopic study and electrochemical characterization. *Journal of Materials Chemistry* **11**, 1378-1382 (2001).

22 Si, W. *et al.* Electrochemical sensing of acetaminophen based on poly (3, 4-ethylenedioxythiophene)/graphene oxide composites. *Sensors and Actuators B: Chemical* **193**, 823-829 (2014).
23 Wang, M. *et al.* A wearable electrochemical biosensor for the monitoring of metabolites and nutrients. *Nature Biomedical Engineering* **6**, 1225-1235 (2022).
24 Tamilselvi, V., Sribalaji, S., Vigneshwaran, P., Vinu, P. & GeethaRamani, J. in *2020 6th International conference on advanced computing and communication systems (ICACCS).* 386-389 (IEEE).
25 Coskun, V., Ozdenizci, B. & Ok, K. A survey on near field communication (NFC) technology. *Wireless personal communications* **71**, 2259-2294 (2013).
26 He, Z. *et al.* Highly stretchable, deformation-stable wireless powering antenna for wearable electronics. *Nano Energy* **112**, 108461 (2023).
27 Bandodkar, A. J. *et al.* Battery-free, skin-interfaced microfluidic/electronic systems for simultaneous electrochemical, colorimetric, and volumetric analysis of sweat. *Science advances* **5**, eaav3294 (2019).
28 Zhang, J. *et al.* Flexible graphene-assembled film-based antenna for wireless wearable sensor with miniaturized size and high sensitivity. *ACS omega* **5**, 12937-12943 (2020).
29 Escobedo, P., Bhattacharjee, M., Nikbakhtnasrabadi, F. & Dahiya, R. Smart bandage with wireless strain and temperature sensors and batteryless NFC tag. *IEEE Internet of Things Journal* **8**, 5093-5100 (2020).
30 Sindha, D. & Raval, F. Study of Liquid Crystal Polymer Based Flexible Antenna. *International Journal of Advanced Research in Computer Engineering & Technology (IJARCET)* **5**, 1-6 (2016).
31 Hajiaghajani, A. *et al.* Textile-integrated metamaterials for near-field multibody area networks. *Nature Electronics* **4**, 808-817 (2021).
32 Liu, Y. *et al.* Synthesis and applications of low dielectric polyimide. *Resources Chemicals and Materials* **2**, 49-62 (2023).
33 Zareei, A. *et al.* Highly conductive copper–silver bimodal paste for low-cost printed electronics. *ACS Applied Electronic Materials* **3**, 3352-3364 (2021).
34 Maina, M. R. *et al.* Influence of surface state in micro-welding of copper by Nd: YAG laser. *Applied Sciences* **8**, 2364 (2018).
35 Myny, K. *et al.* in *2017 IEEE International Solid-State Circuits Conference (ISSCC).* 258-259 (IEEE).
36 Faisal, M. *et al.* in *2019 1st International Conference on Advances in Science, Engineering and Robotics Technology (ICASERT).* 1-5 (IEEE).
37 Chatterjee, S., Thakur, R. S., Yadav, R. N., Gupta, L. & Raghuvanshi, D. K. Review of noise removal techniques in ECG signals. *IET Signal Processing* **14**, 569-590 (2020).
38 Kher, R. Signal processing techniques for removing noise from ECG signals. *J. Biomed. Eng. Res* **3**, 1-9 (2019).
39 Berwal, D., V, C. R., Dewan, S., J, C. V. & Baghini, M. S. Motion Artifact Removal in Ambulatory ECG Signal for Heart Rate Variability Analysis. *IEEE Sensors Journal* **19**, 12432-12442 (2019).
40 Pan, J. & Tompkins, W. J. A Real-Time QRS Detection Algorithm. *IEEE Transactions on Biomedical Engineering* **BME-32**, 230-236 (1985).
41 Strasser, F., Muma, M. & Zoubir, A. M. in *2012 Proceedings of the 20th European Signal Processing Conference (EUSIPCO).* 899-903 (IEEE).
42 Mejía-Mejía, E. & Kyriacou, P. A. Effects of noise and filtering strategies on the extraction of pulse rate variability from photoplethysmograms. *Biomedical Signal Processing and Control* **80**, 104291 (2023).
43 Dall'Olio, L. *et al.* Prediction of vascular aging based on smartphone acquired PPG signals. *Scientific Reports* **10**, 19756 (2020).
44 Goda, M. A., Charlton, P. H. & Behar, J. A. pyppg: A python toolbox for comprehensive photoplethysmography signal analysis. *arXiv preprint arXiv:2309.13767* (2023).
45 Critchley, H. & Nagai, Y. Electrodermal activity (EDA). *Encyclopedia of behavioral medicine* **78**, 666-669 (2013).

46 Kleckner, I. R. *et al.* Simple, transparent, and flexible automated quality assessment procedures for ambulatory electrodermal activity data. *IEEE Transactions on Biomedical Engineering* **65**, 1460-1467 (2017).
47 Braithwaite, J. J., Watson, D. G., Jones, R. & Rowe, M. A guide for analysing electrodermal activity (EDA) & skin conductance responses (SCRs) for psychological experiments. *Psychophysiology* **49**, 1017-1034 (2013).
48 Lu, Z. & Ozek, B. Transformer encoder with multiscale deep learning for pain classification using physiological signals. *Frontiers in Physiology* **14**, 1294577 (2023).
49 Khan, A., Sohail, A., Zahoora, U. & Qureshi, A. S. A survey of the recent architectures of deep convolutional neural networks. *Artificial intelligence review* **53**, 5455-5516 (2020).
50 Szegedy, C. *et al.* in *2015 IEEE Conference on Computer Vision and Pattern Recognition (CVPR).* 1-9.
51 Szegedy, C. *et al.* in *Proceedings of the IEEE conference on computer vision and pattern recognition.* 1-9.
52 Mou, L. *et al.* Multimodal driver distraction detection using dual-channel network of CNN and Transformer. *Expert Systems with Applications* **234**, 121066 (2023).
53 Kingma, D. P. & Ba, J. Adam: A method for stochastic optimization. *arXiv preprint arXiv:1412.6980* (2014).
54 Kim, H.-G., Cheon, E.-J., Bai, D.-S., Lee, Y. H. & Koo, B.-H. Stress and heart rate variability: a meta-analysis and review of the literature. *Psychiatry investigation* **15**, 235 (2018).
55 Gedam, S. & Paul, S. A review on mental stress detection using wearable sensors and machine learning techniques. *IEEE Access* **9**, 84045-84066 (2021).
56 Charlton, P. H., Celka, P., Farukh, B., Chowienczyk, P. & Alastruey, J. Assessing mental stress from the photoplethysmogram: a numerical study. *Physiological measurement* **39**, 054001 (2018).
57 Ganapathy, N., Veeranki, Y. R. & Swaminathan, R. Convolutional neural network based emotion classification using electrodermal activity signals and time-frequency features. *Expert Systems with Applications* **159**, 113571 (2020).
58 Marazziti, D., Di Muro, A. & Castrogiovanni, P. Psychological stress and body temperature changes in humans. *Physiology & behavior* **52**, 393-395 (1992).
59 Wang, C. *et al.* High-precision flexible sweat self-collection sensor for mental stress evaluation. *npj Flexible Electronics* **8**, 47 (2024).
60 Xu, C. *et al.* A physicochemical-sensing electronic skin for stress response monitoring. *Nature Electronics* **7**, 168-179 (2024).
61 Hossain, N. I., Noushin, T. & Tabassum, S. StressFit: A hybrid wearable physicochemical sensor suite for simultaneously measuring electromyogram and sweat cortisol. *Scientific Reports* **14**, 29667 (2024).
62 Jang, H. *et al.* Graphene e-tattoos for unobstructive ambulatory electrodermal activity sensing on the palm enabled by heterogeneous serpentine ribbons. *Nature Communications* **13**, 6604 (2022).
63 Kim, H. *et al.* Fully integrated, stretchable, wireless skin-conformal bioelectronics for continuous stress monitoring in daily life. *Advanced Science* **7**, 2000810 (2020).
64 Ding, Y. *et al.* Integrated mental stress smartwatch based on sweat cortisol and HRV sensors. *Biosensors and Bioelectronics* **265**, 116691 (2024).
65 Wang, B. *et al.* Wearable aptamer-field-effect transistor sensing system for noninvasive cortisol monitoring. *Science advances* **8**, eabk0967 (2022).
