# Supplementary material for: A quantitative, multimodal wearable bioelectronic device for comprehensive stress assessment and sub-classification
Source: Nat Commun. 2026 Jan 29;17:1150. doi: 10.1038/s41467-025-67747-9 (PMC12858848; doi:10.1038/s41467-025-67747-9)
Supplement: Supplementary file 2 — Description of Additional Supplementary Files [file 41467_2025_67747_MOESM2_ESM.pdf]

Description of Additional Supplementary Files

Supplementary Movie 1: Flexible microfluidic channel infilling demonstration (ex-situ) for the MSB patch.

Supplementary Movie 2: Simulation and exsitu validation of microfluidic channel refreshing with sequential dyes.

Supplementary Movie 3: In-situ (on-body) sweat sample generation and channel infilling using IP-integrated microfluidics.

Supplementary Movie 4: In-situ (on-body) wireless IP-based sweat generation using the SQC-SAS module.

Supplementary Movie 5: On-demand, realtime MSB and PSI biosignal collection via smartphone-powered SQC-SAS (with silicone case).

Supplementary Movie 6: Prolonged continuous in-situ MSB and PSI biosignal collection via the WLR wireless reader (with silicone case).

Supplementary Movie 7: On-demand, realtime MSB and PSI biosignal collection via SQC-SAS without the silicone case.

Supplementary Movie 8: Prolonged continuous in-situ MSB and PSI biosignal collection via the WLR wireless reader without the silicone case.

Supplementary Movie 9: Ex-situ demonstration of the regeneration capability of the MSB patch for repeated cortisol sensing.

Supplementary Movie 10: In-situ (on-body) demonstration of the regeneration capability of the MSB patch during continuous PSI and MSB monitoring.

Supplementary Movie 11: Demonstration of SQC-SAS stress assessment using MSB and PSI biosignals before, during, and after controlled stress.
